# A PI(3,5)P_2_ reporter reveals PIKfyve activity and dynamics on macropinosomes and phagosomes

**DOI:** 10.1083/jcb.202209077

**Published:** 2023-06-29

**Authors:** James H. Vines, Hannes Maib, Catherine M. Buckley, Aurelie Gueho, Zhou Zhu, Thierry Soldati, David H. Murray, Jason S. King

**Affiliations:** 1 School of Biosciences, University of Sheffield, Firth Court Western Bank, Sheffield, UK; 2 Division of Molecular, Cell and Developmental Biology, School of Life Sciences, University of Dundee, Dundee, UK; 3 Department of Biochemistry, Faculty of Science, University of Geneva, Geneva, Switzerland

## Abstract

Phosphoinositide signaling lipids (PIPs) are key regulators of membrane identity and trafficking. Of these, PI(3,5)P_2_ is one of the least well-understood, despite key roles in many endocytic pathways including phagocytosis and macropinocytosis. PI(3,5)P_2_ is generated by the phosphoinositide 5-kinase PIKfyve, which is critical for phagosomal digestion and antimicrobial activity. However PI(3,5)P_2_ dynamics and regulation remain unclear due to lack of reliable reporters. Using the amoeba *Dictyostelium discoideum,* we identify SnxA as a highly selective PI(3,5)P_2_-binding protein and characterize its use as a reporter for PI(3,5)P_2_ in both *Dictyostelium* and mammalian cells. Using GFP-SnxA, we demonstrate that *Dictyostelium* phagosomes and macropinosomes accumulate PI(3,5)P_2_ 3 min after engulfment but are then retained differently, indicating pathway-specific regulation. We further find that PIKfyve recruitment and activity are separable and that PIKfyve activation stimulates its own dissociation. SnxA is therefore a new tool for reporting PI(3,5)P_2_ in live cells that reveals key mechanistic details of the role and regulation of PIKfyve/PI(3,5)P_2_.

## Teaser

In this work, the authors describe a new biosensor for the phosphoinositide signaling lipid PI(3,5)P_2_. They demonstrate that this reporter faithfully reports PI(3,5)P_2_ dynamics in both *Dictyostelium* amoebae and mammalian cells and uncover details of how PI(3,5)P_2_ formation is regulated during phagosome maturation.

## Introduction

The inositol phospholipids are a family of interconvertible signaling molecules that define distinct endocytic compartments. Differential phosphorylation at three positions on the inositol headgroup allows eight different signaling species to be generated. These are used to recruit specific effector proteins to membranes and are interconverted by a large family of lipid kinases and phosphatases. Transitions between different phosphoinositides (PIPs) are thus central components of the identity, sorting, and progression of almost all membrane compartments.

One of the least understood PIP transitions is the generation of PI(3,5)P_2_ by the phosphoinositide 5-kinase PIKfyve (Fab1p in yeast). PIKfyve is recruited to early endosomes via its FYVE (Fab1, YOTB, Vac1, and EEA1) domain that binds PI(3)P and subsequently phosphorylates it at the 5-position to produce PI(3,5)P_2_ (Cabezas et al., 2006; Sbrissa et al., 1999; Yamamoto et al., 1995). PIKfyve is in a complex with the scaffolding regulator Vac14 (ArPIKfyve) and the 5-phosphatase Fig4 (Sac3), which catalyzes the reverse reaction (Botelho et al., 2008; Rudge et al., 2004; Sbrissa et al., 2007). This complex is under intricate regulation, and while genetic, biochemical, and structural studies have provided some mechanistic insight (Berwick et al., 2004; Ikonomov et al., 2009; Lees et al., 2020; Sbrissa et al., 2000), the dynamics of PI(3,5)P_2_ formation in vivo have remained elusive due to the lack of a reliable live-cell reporter.

Disruption of PIKfyve causes severe and widespread defects in endocytic trafficking in all organisms studied, typified by the formation of swollen endosomes and defects in lysosomal degradation (Buckley et al., 2019; Choy et al., 2018; de Lartigue et al., 2009; Dove et al., 2009; Ikonomov et al., 2001; Kim et al., 2014; Krishna et al., 2016; Nicot et al., 2006). PIKfyve activity is therefore critical for several physiologically important pathways such as the digestion of autophagic and phagocytic cargo and endocytosed receptors (Buckley et al., 2019; de Lartigue et al., 2009; Demirsoy et al., 2017; Ferguson et al., 2009; Kim et al., 2014; McCartney et al., 2014b; Rusten et al., 2006). Unsurprisingly, disrupted PI(3,5)P_2_ signaling is implicated in a broad range of diseases (reviewed in McCartney et al., 2014a; Shisheva, 2012).

How these phenotypes result from PI(3,5)P_2_ deficiency is not clear. Several studies have indicated that PI(3,5)P_2_ can directly regulate lysosomal ion channels such as mucolipin (TRPML), two-pore calcium channels, and the chloride channel ClC7 (Dong et al., 2010; Leray et al., 2022; Samie et al., 2013; Wang et al., 2012). PI(3,5)P_2_ has also been shown to regulate association of the two vacuolar ATPase subcomplexes on the yeast vacuole, although this has not yet been confirmed in other organisms (Li et al., 2014). Loss of PI(3,5)P_2_ can also affect lysosomal transport and delivery to the compartments targeted for degradation (Li et al., 2016). Each mechanism can potentially contribute to the phenotypes observed, but our understanding is limited by poor knowledge of where and when PI(3,5)P_2_ is generated.

We and others have previously demonstrated a key and evolutionarily conserved role for PIKfyve during phagosome maturation. Phagocytosis is used by cells to engulf large extracellular particles such as microbes, dead cells, and debris, and is highly related to the process of macropinocytosis, whereby cells take up bulk extracellular fluid (King and Kay, 2019). After engulfment, both pathways follow a complex series of maturation steps to deliver antimicrobial and digestive components to defend against potential pathogens, recycle nutrients, or generate antigens for presentation (Buckley et al., 2016; Chen et al., 2010; Levin et al., 2016).

PIKfyve is important for phagosome–lysosome fusion in macrophages, neutrophils, and the amoeba *Dictyostelium discoideum* (Buckley et al., 2019; Dayam et al., 2015, 2017; Isobe et al., 2019; Kim et al., 2014). We recently showed that loss of PIKfyve in *Dictyostelium* severely disrupts phagosomal proteolysis and killing, rendering them hypersensitive to infection with *Legionella pneumophila* (Buckley et al., 2019). Whilst this demonstrates the importance of PIKfyve in innate immunity, its regulation remains poorly understood.

A major obstacle to understanding PI(3,5)P_2_ function and regulation has been the lack of a reliable reporter to visualize when and where it is generated in cells. Although a tandem repeat of the N-terminal lipid-binding region of TRPML showed PI(3,5)P_2_ specificity in vitro and looked promising in cells (Li et al., 2013), others found clear evidence of non-specific endosomal recruitment and retention after PI(3,5)P_2_ depletion (Hammond et al., 2015). We therefore set out to identify and validate an alternative reporter to faithfully describe PI(3,5)P_2_ dynamics within live cells. Here, we identify and characterize *Dictyostelium* SnxA as a highly selective PI(3,5)P_2_-binding protein. Using phagosome and macropinosomes maturation as accessible endocytic pathways, we use fluorescent SnxA fusions as reporters to visualize and describe PI(3,5)P_2_ dynamics in live cells for the first time and uncover key regulatory steps in both PIKfyve recruitment and activity.

## Results

### Identification of SnxA as a PI(3,5)P_2_-binding protein in *Dictyostelium*

To identify a specific PI(3,5)P_2_-binding protein that could be used as a reporter, we started with the assumption that such proteins would only be recruited to membranes in the presence of PIKfyve. As PX (Phox homology) domains are frequently used by proteins to bind specific PIPs, we searched the *Dictyostelium* genome for proteins containing a PX domain and screened C-terminally tagged GFP fusions of these for PIKfyve-dependent localization by expression in both WT and ΔPIKfyve cells.

**Figure 1.**
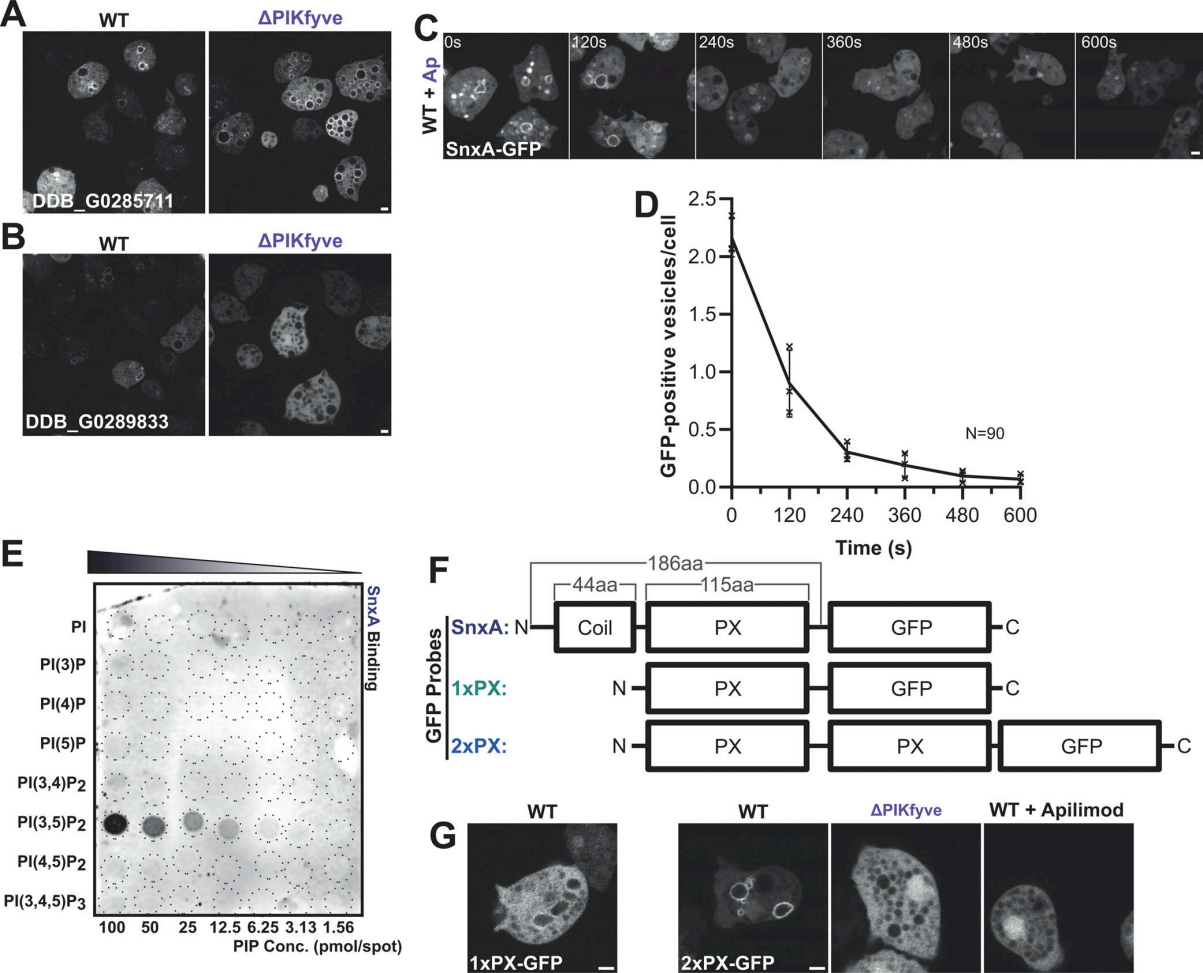
**Identification of SnxA as a PI(3,5)P**_**2**_ **reporter. (A and B)** Expression of GFP-fusions of PX-domain-containing proteins in WT and ΔPIKfyve Ax3 *Dictyostelium*. DDB_G0285711 (A) localized to large vesicles in both cells, whereas DDB_G0289833 (B) was completely cytosolic in ΔPIKfyve. **(C)** Timelapse of DDB_G0289833 (SnxA)-GFP localization in Ax2 cells after addition of the PIKfyve inhibitor apilimod (3 μM). **(D)** Quantification of the number of SnxA-GFP vesicles per cell. >90 cells measured per time-point; data show mean ± SEM of three independent experiments. **(E)** Representative PIP array using lysates from WT cells expressing SnxA-GFP and probed with anti-GFP antibody. **(F)** Schematics of SnxA-GFP, 1xPX-GFP, and 2xPX-GFP. **(G)** Representative images of their localization in the cells indicated. 1xPX-GFP remained predominately cytosolic in WT cells, whereas 2xPX-GFP localized strongly to large vesicles, which was lost in ΔPIKfyve and apilimod treated cells. All scale bars = 2 μm. Source data are available for this figure: SourceData F1.

Of the proteins cloned, most localized to membranes irrespective of the presence or absence of PIKfyve (e.g., Fig. 1 A). However, one (DDB_G0289833) clearly localized to large vesicles with the appearance of macropinosomes in WT but was completely cytosolic in ΔPIKfyve cells (Fig. 1 B). DDB_G0289833 is previously unstudied and contains a coiled-coil domain in addition to the PX domain. As this domain combination is found in many mammalian sorting nexins, we named the *Dictyostelium* protein SnxA. To further confirm the dependence of SnxA recruitment on PIKfyve, we applied the PIKfyve inhibitor apilimod (Cai et al., 2013) to WT cells expressing SnxA-GFP. This caused rapid dissociation of SnxA-GFP from all membranes, becoming completely cytosolic within 10 min (Fig. 1, C and D).

PIKfyve activity is required for the formation of both PI(3,5)P_2_ and PI(5)P. To determine the lipid-binding specificity of SnxA, we examined binding to PIP arrays using lysates from SnxA-GFP expressing *Dictyostelium* cells. In this assay, SnxA-GFP bound with at least a 20-fold preference to PI(3,5)P_2_ over all other PIPs with almost no non-specific binding at even the highest lipid concentrations (Fig. 1 E).

**Figure S1.**
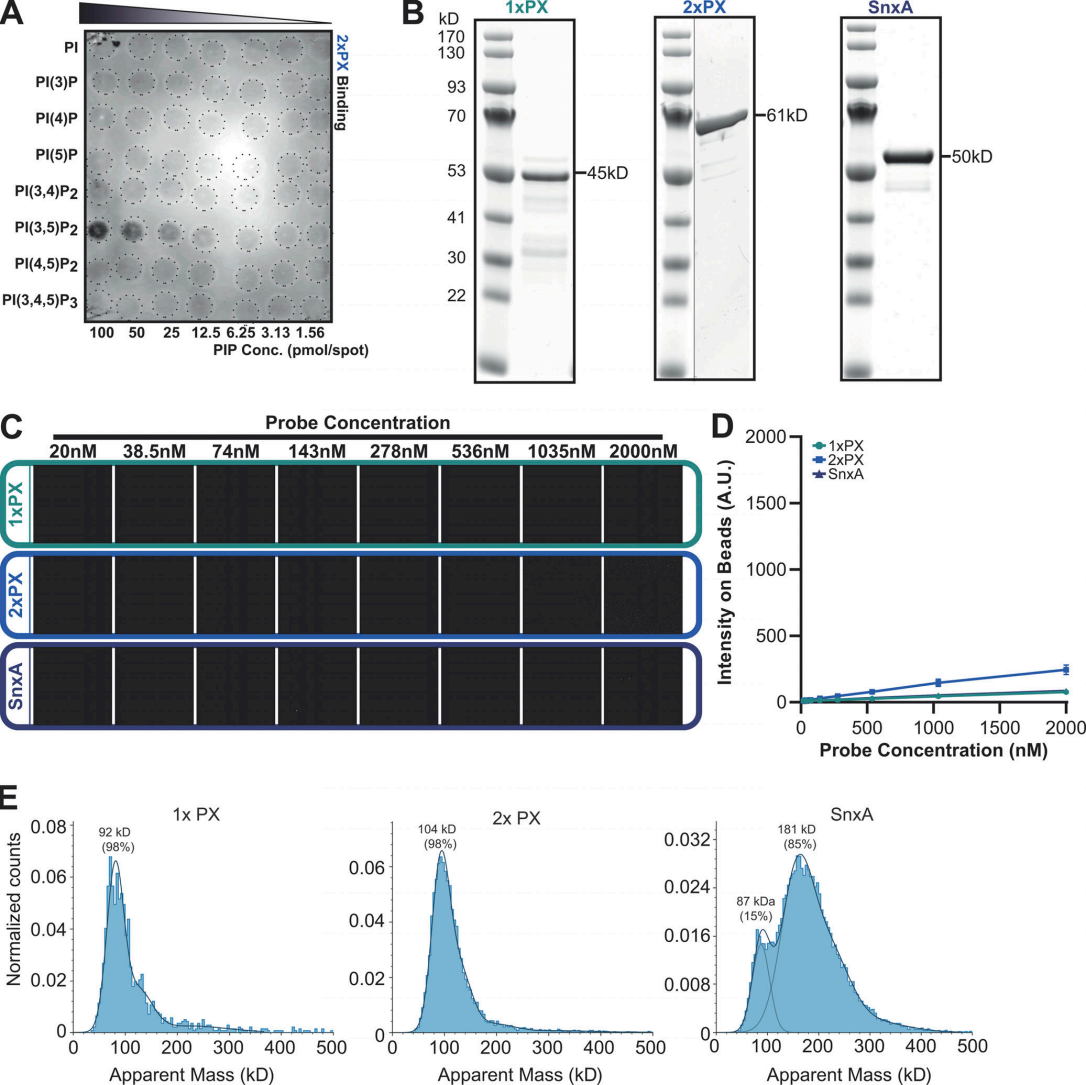
**SnxA does not bind PI(5)P. (A)** PIP array performed using lysates from WT cells expressing 2xPX-GFP and probed with anti-GFP antibody. **(B)** Coomassie-stained SDS-PAGE gels of recombinant GFP fusion SnxA probes used for lipid binding assays. **(C)** Representative images of SnxA probe titrated against PI(5)P-containing membrane-coated beads. **(D)** This is quantified and plotted as the mean ± SD from three independent experiments. **(E)** Mass photometry analysis of recombinant SnxA fusions. Note that exact molecular weights of native proteins are inaccurate below ∼100 kD using this technique. Source data are available for this figure: SourceData FS1.

To optimize lipid binding and reporter characteristics, we then tested GFP-fusions of either the PX domain alone or a tandem repeat of this domain (1xPX and 2xPX-GFP, respectively, Fig. 1 F). 1xPX-GFP had low contrast, localizing poorly to large vesicles in some cells, but was completely cytosolic in many others. Contrast was significantly enhanced in the 2xPX construct, which localized with similar characteristics and PIKfyve-dependence as full-length SnxA-GFP (Fig. 1 G). The 2xPX-GFP construct also bound similarly to PIP arrays, localizing specifically to PI(3,5)P_2_ (Fig. S1 A).

**Figure 2.**
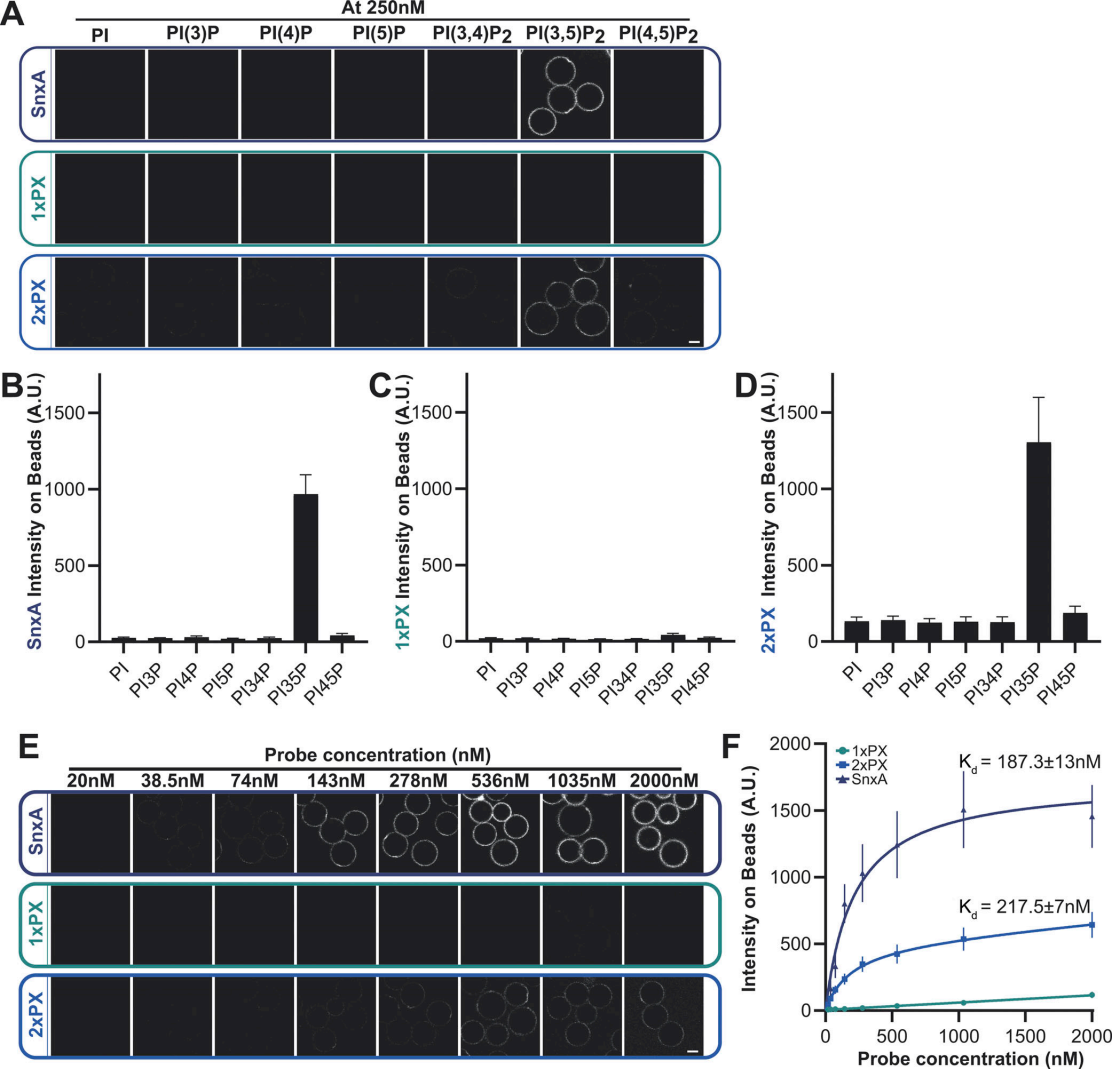
**PI(3,5)P**_**2**_ **binding specificity. (A–D)** Representative images and quantification of binding of recombinant GFP-SnxA probes to membrane-coated beads containing the indicated PIPs. **(E)** Binding of each SnxA probe titrated against beads harboring PI(3,5)P_2_. **(F)** Intensities measured under identical imaging conditions and apparent K_d_s. All graphs show the mean ± SD from three independent experiments; scale bars indicate 5 μm.

To confirm PI(3,5)P_2_ specificity using more physiologically relevant membranes, all three GFP fusions were produced recombinantly (Fig. S1 B) and screened for binding to beads coated with bilayers consisting of 5% phosphoinositide and 95% 1-palmitoyl-2-oleoyl-sn-glycero-3-phosphocholine. Lipid binding and affinities were then determined by measuring GFP recruitment by microscopy (Maib and Murray, 2022). As anticipated, both SnxA-GFP and 2xPX-GFP probes showed high specificity for PI(3,5)P_2_, while 1xPX showed very little binding (Fig. 2, A–D). By titrating the probe concentration at a fixed amount of membrane, we determined an apparent K_d_ of 187.3 ± 13 nM for full-length SnxA and 217.5 ± 7 nM for 2xPX (Fig. 2, E and F). Importantly, no binding to PI(5)P, the other product of PIKfyve, was observed under any conditions (Fig. S1, C and D).

These data demonstrate that the PX domain of SnxA is a highly selective PI(3,5)P_2_-binding domain. Although a tandem repeat of this domain increases binding affinity, this unexpectedly provides no improvement over the full-length protein. We therefore tested whether the full-length protein dimerizes by measuring the mass distribution of the recombinant fusion proteins by mass photometry (Sonn-Segev et al., 2020). While the 1x and 2xPX GFP fusions existed as a single population with apparent molecular weights of 92 and 104 kD, respectively, 85% of the full-length SnxA protein had an apparent molecular weight of 181 kD—approximately double that expected for a monomer. Full-length SnxA likely dimerizes via its coiled-coil domain, providing an avidity similar to the 2xPX construct. This may be advantageous for imaging as it will incorporate two GFP-moieties into each dimer and increase brightness, although it is also possible that full-length SnxA could dimerize with other proteins within cells.

### Validation of SnxA as a PI(3,5)P2 reporter in mammalian cells

Our data in *Dictyostelium* indicate that SnxA has high selectivity for both PI(3,5)P2 and reversible binding, indicated by rapid dissociation upon PIKfyve inhibition. To test whether it could be universally used as a PI(3,5)P_2_ reporter in other organisms, we made human-codon-optimized versions of both the full-length SnxA and the 2xPX construct to express as N-terminal GFP-fusions in a panel of mammalian cell lines.

**Figure 3.**
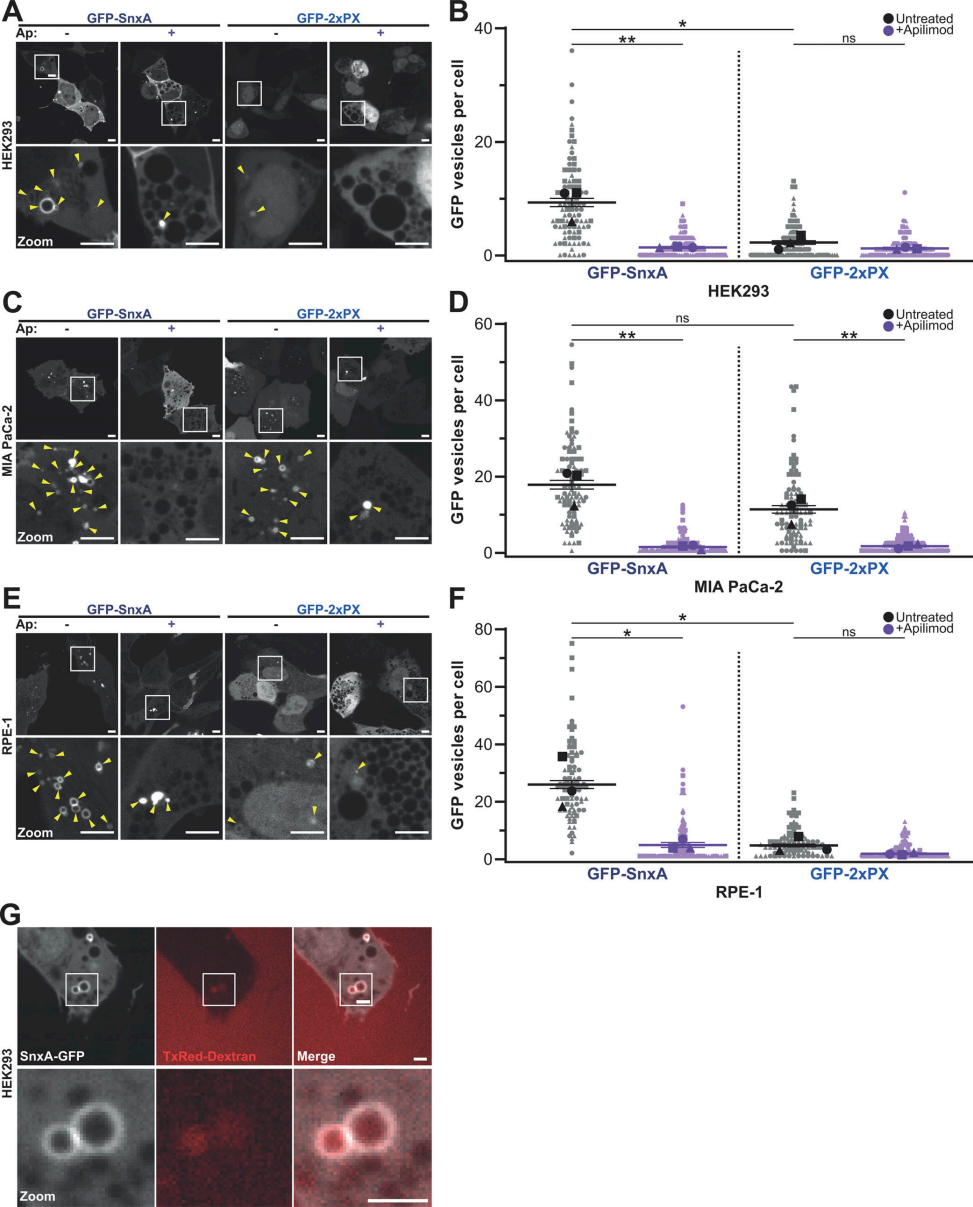
**Expression of SnxA fusions in mammalian cells. (A–F)** Representative images and quantification of either full-length SnxA or 2xPX GFP fusions expressed in either HEK293, MIA PaCa-2, or RPE-1 cells. Top panels of A, C, and E are maximum intensity projections of a spinning-disc Z-stack, with single sections and enlargements of the boxed regions below. Cells were either left untreated (−) or pretreated with 1 μM apilimod (Ap; +) for 1 h prior to imaging. Arrowheads indicate the GFP-positive structures that were quantified over three independent experiments in B, D, and F. 30 cells per experiment for three independent experiments were scored for each condition and P values were calculated by unpaired *T* test (*n* = 3, *P < 0.05, **P < 0.01); scale bars represent 10 μm. **(G)** Shows an image from a timeseries (Video 1) of a HEK293 cell expressing GFP-SnxA just after addition of Texas-Red dextran. Scale bars represent 2 μm.

In all cell lines examined, both full-length SnxA and 2xPX GFP fusions localized clearly to large intracellular vesicles (Fig. 3). The localization was almost completely lost upon inhibition of PIKfyve with apilimod, and although a small number of GFP-positive puncta were observed in some cells, these were much smaller and resembled aggregates, with no visible lumen (see Fig. 3, A, C and E, zoom panels). Effective inhibition of PIKfyve was confirmed by the presence of typically swollen vacuoles visible in these cells.

**Figure S2.**
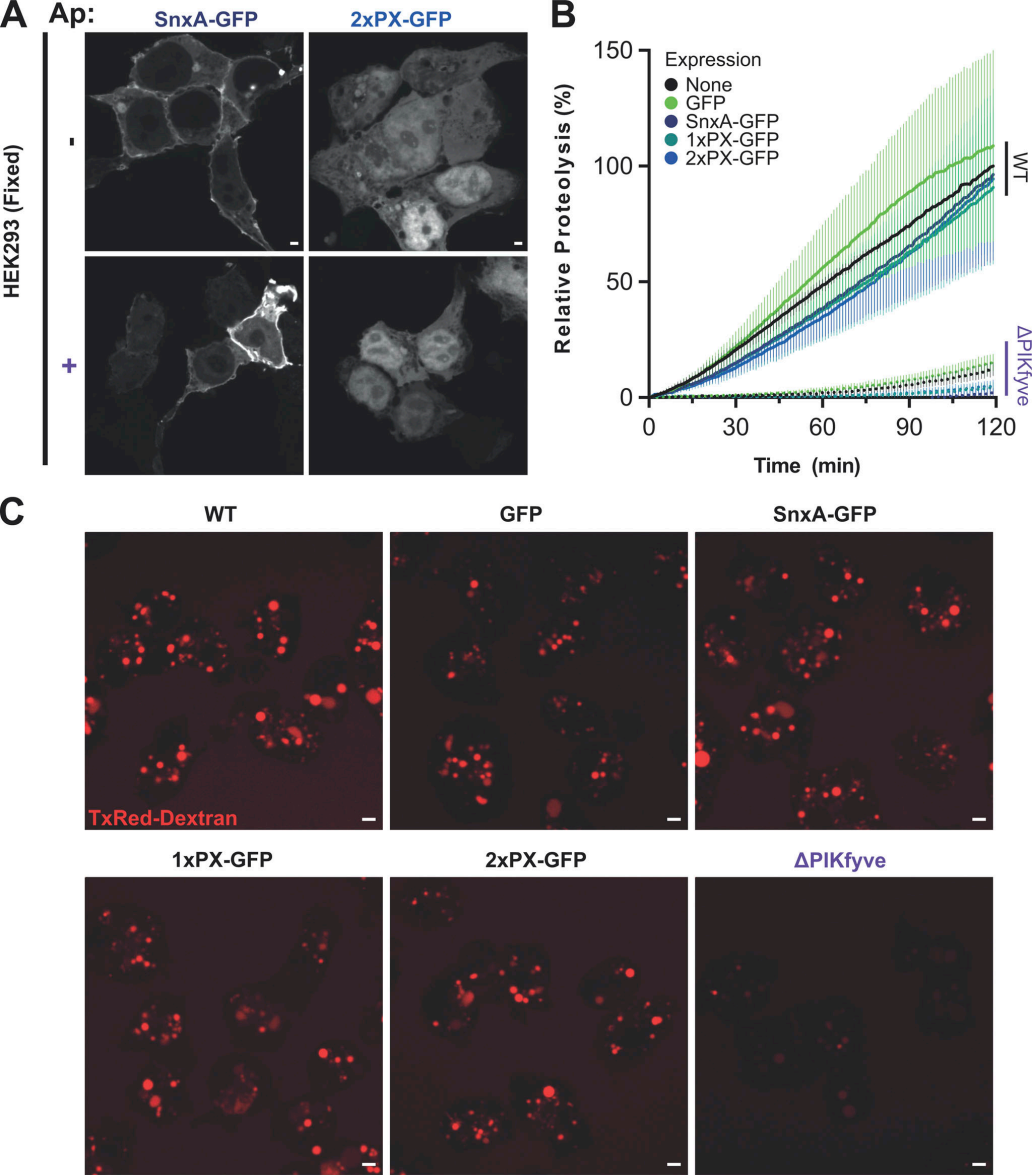
**Characterization of SnxA probes. (A)** Confocal images of HEK293 cells transfected with either full-length or 2xPX-GFP fusions ± apilimod after fixation with paraformaldehyde. **(B)** Phagosomal proteolysis of both Ax2 (WT) and ΔPIKfyve *Dictyostelium* cells expressing different SnxA-derived probes, measured by digestion of bead-conjugated DQ-BSA. Data show mean ± SD of three independent experiments. **(C)** Images of macropinosomes in Ax2 cells expressing each probe or ΔPIKfyve cells after overnight incubation in Texas-Red dextran. The bright vesicles indicate concentration of macropinosomes contents. All scale bars represent 2 μm.

We also noted that upon paraformaldehyde fixation, GFP-SnxA was also partly enriched near the plasma membrane (Fig. S2 A). This was insensitive to apilimod treatment and did not occur with the GFP-2xPX probe. A similar localization was also never visible in unfixed cells (e.g., Fig. 3). Plasma membrane enrichment is therefore due to non-specific binding via the coiled-coil domain rather than an additional pool of PI(3,5)P_2_. As this localization is sufficiently distinct from endosomal PI(3,5)P_2_ pools it does not prevent use of GFP-SnxA as a probe for experiments where fixation is necessary; however, we advocate caution interpreting any observations at or near the cell surface and the use of appropriate controls.

Although both fusion proteins were recruited to vesicles of comparable size, significantly fewer structures were often observed in cells expressing GFP-2xPX than those expressing the full-length GFP-SnxA. This varied between cell lines; both reporters behaved similarly in MIA PaCa-2 pancreatic cancer cells, whereas the 2xPX-GFP was recruited to very few structures in both retinal pigment epithelial (RPE-1) and human embryonic kidney (HEK293) cells. As some cell lines were unaffected, the differences in others must lie in negative effects of reporter expression rather than different PI(3,5)P2 binding dynamics. We also noticed that cells expressing GFP-2xPX grew more slowly and generally appeared less healthy than untransfected or GFP-SnxA–expressing cells. Therefore, while a tandem PX domain construct may be a less complex reporter for PI(3,5)P_2_, it has a greater potential to disrupt cellular function than full-length SnxA. The degree of disruption will depend on factors such as expression levels and cell type and should be assessed for each specific experimental context, as for all such probes.

As the large vesicles observed resemble macropinosomes, we tested this by incubating HEK293 cells with 70 kD Texas-Red dextran, which is predominantly taken up by this route. Dextran clearly labeled all GFP-SnxA positive vesicles, indicating that they are macropinocytic in origin. However, not all dextran-containing vesicles had GFP-SnxA, indicating that PI(3,5)P_2_ is only transiently present (Fig. 3 G and Video 1). Although PI(3,5)P_2_ is thought to be a general component of late endosomal/lysosomal pathways, we did not observe small puncta that could represent classical endocytic compartments. See the discussion for potential explanations.

**Video 1.** **Macropinocytosis in HEK293 cells expressing GFP-SnxA (white) directly following addition of 2.5 mg/ml Texas-Red dextran (red), related to** Fig. 3 G**.** Images were captured every 60 s by spinning-disc microscopy and displayed at four frames per second. Scale bar = 2 μm.

### SnxA-probes do not disrupt PIKfyve signaling in *Dictyostelium*

To validate SnxA as a PI(3,5)P_2_ reporter in detail we returned to the *Dictyostelium* model system. As disruption of PIKfyve blocks both phagosomal proteolysis and the concentration of macropinosome content, we started by testing whether expression of SnxA-GFP fusions also affected these processes (Buckley et al., 2019). Neither full-length SnxA, 1xPX, nor 2xPX GFP-fusions had deleterious effects in either assay, and we did not notice any obvious changes to cell morphology or growth (Fig. S2, C and D). Therefore none of the SnxA probes appear to disrupt PI(3,5)P_2_ signaling in *Dictyostelium*.

**Figure S3.**
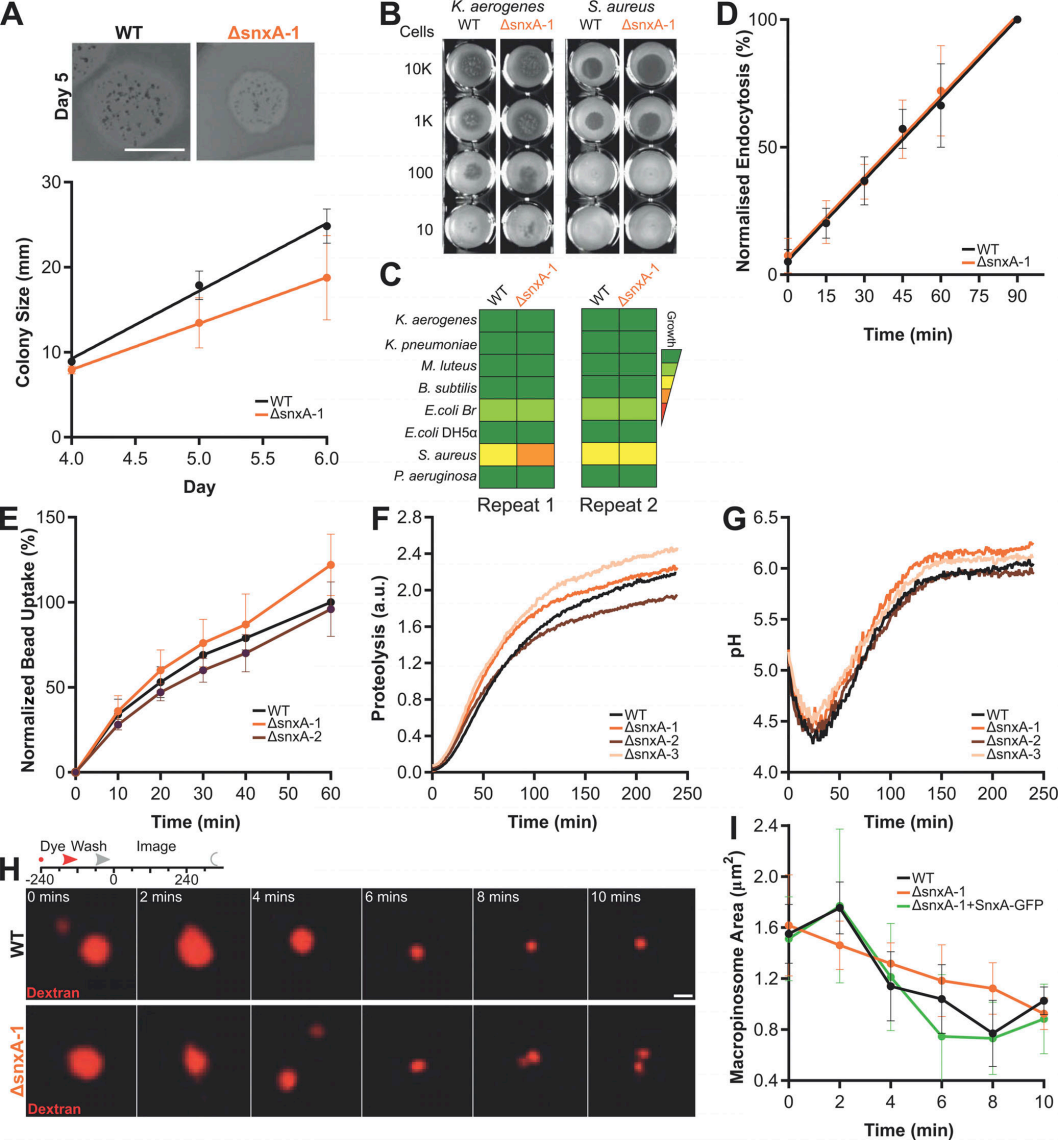
**Loss of SnxA has little effect on phagosome or macropinosome maturation. (A)** Images of WT and ΔsnxA-1 colonies growing on a lawn of *K. aerogenes*. Average colony diameter over time is shown below. Scale bar = 10 mm. **(B)** Growth of mutant cells in different bacteria. Numbers indicate the number of *Dictyostelium* cells seeded on each well. **(C)** Summary of growth on a range of bacteria screened as in B. **(D)** Quantification of endocytosis calculated by FITC dextran uptake. **(E)** Phagocytosis of 1 μm fluorescent beads, measured by flow cytometry and normalized to WT cells at 60 min. **(F and G)** Phagosomal proteolysis (F) and pH (G) over time, measured by fluorescence changes after engulfment of either DQ-BSA or FITC/Alexa594 reporter beads, respectively. **(H)** Representative images of macropinosomes shrinking after a 2-min (mins) pulse of TRITC-dextran; scale bar = 2 μm. **(I)** Size is quantified. All data shown are the mean ± SD of at least three independent experiments. ΔsnxA-1, 2, and 3 indicate independent knockout clones.

We also generated multiple independent *SnxA* knockouts and asked whether SnxA played an important function in phagosome maturation itself. Unlike ΔPIKfyve cells, ΔSnxA mutants had no significant defects in bacterial growth, bulk fluid uptake, phagocytosis, phagosome proteolysis, or acidification (Fig. S3, A–G). Following an extensive investigation, the only difference we could observe between WT and ΔSnxA cells was a subtle but reproducible alteration in the dynamics of macropinosome shrinkage after internalization: whilst macropinosomes in WT cells transiently enlarge after internalization before shrinking and concentrating their content, the expansion phase does not occur in ΔSnxA cells and can be rescued by re-expression of SnxA-GFP (Fig. S3, H and I). Despite this, bulk fluid uptake is unaffected and no axenic growth defect was observed. We speculate that the transient swelling indicates SnxA-dependent fusion of another endocytic compartment to very early macropinosomes, but this requires further investigation. In any case, SnxA is not necessary for maturation of either phagosomes or macropinosomes and, therefore, other effectors of PIKfyve must exist.

### PI(3,5)P_2_ dynamics during phagosome and macropinosome maturation

**Figure 4.**
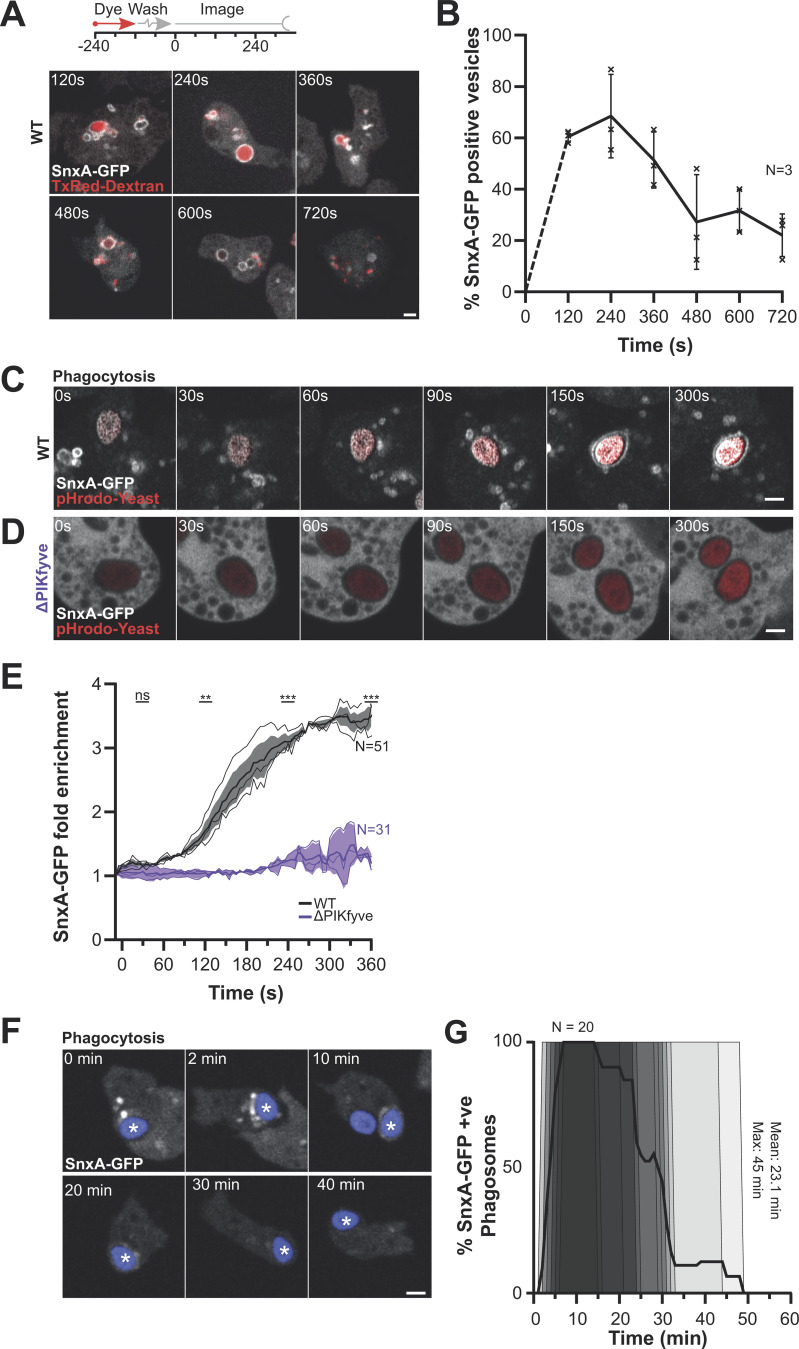
**PI(3,5)P**_**2**_ **dynamics during macropinosome and phagosome maturation. (A)** Recruitment of SnxA-GFP in *Dictyostelium* cells following a 2-min dextran pulse-chase to label macropinosomes. Different fields of view were taken at time points after dextran washout. **(B)** The proportion of macropinosomes positive for SnxA-GFP at each time after dextran addition. >40 vesicles were scored per time point for each of three independent experiments. **(C and D)** Images from timelapse microscopy of SnxA-GFP during phagocytosis of pHrodo-yeast (red) in WT (C) and ΔPIKfyve (D) cells. See Video 2 for the complete sequence. **(E)** Quantification of SnxA-GFP enrichment at the phagosomal membrane averaged over a total of 51 WT and 31 ΔPIKfyve phagosomes across three independent experiments (**P < 0.01, ***P < 0.005. Fold enrichment normalized to 10 s before engulfment. Graph shows the mean ± SEM across independent experiments; thin lines show each the individual repeats. **(F)** Example long-term timelapse of SnxA-GFP recruitment to phagosomes. **(G)** Quantification of 20 phagocytic events followed in this way over three experiments and scored for SnxA-GFP localization over time. All scale bars = 2 μm and all graphs show the mean ± SEM of three independent experiments unless otherwise stated.

To test whether the large SnxA-GFP–positive vesicles observed in *Dictyostelium* were also macropinosomes and determine at which point PI(3,5)P_2_ is present, we labeled macropinosomes with a 2-min pulse of Texas-Red dextran and then imaged different fields of view at time intervals after dextran removal. For optimal imaging and uniform expression, we generated a stably integrated SnxA-GFP cell line selecting for a clone with low expression/high contrast for microscopy experiments. This allows the percentage of macropinosomes positive for GFP to be measured for up to 10 min of maturation (Buckley et al., 2016). This demonstrated that SnxA-GFP is transiently recruited to macropinosomes within 2 min of engulfment, peaking at 4 min before removal after 5–6 min (Fig. 4, A and B).

**Figure S4.**
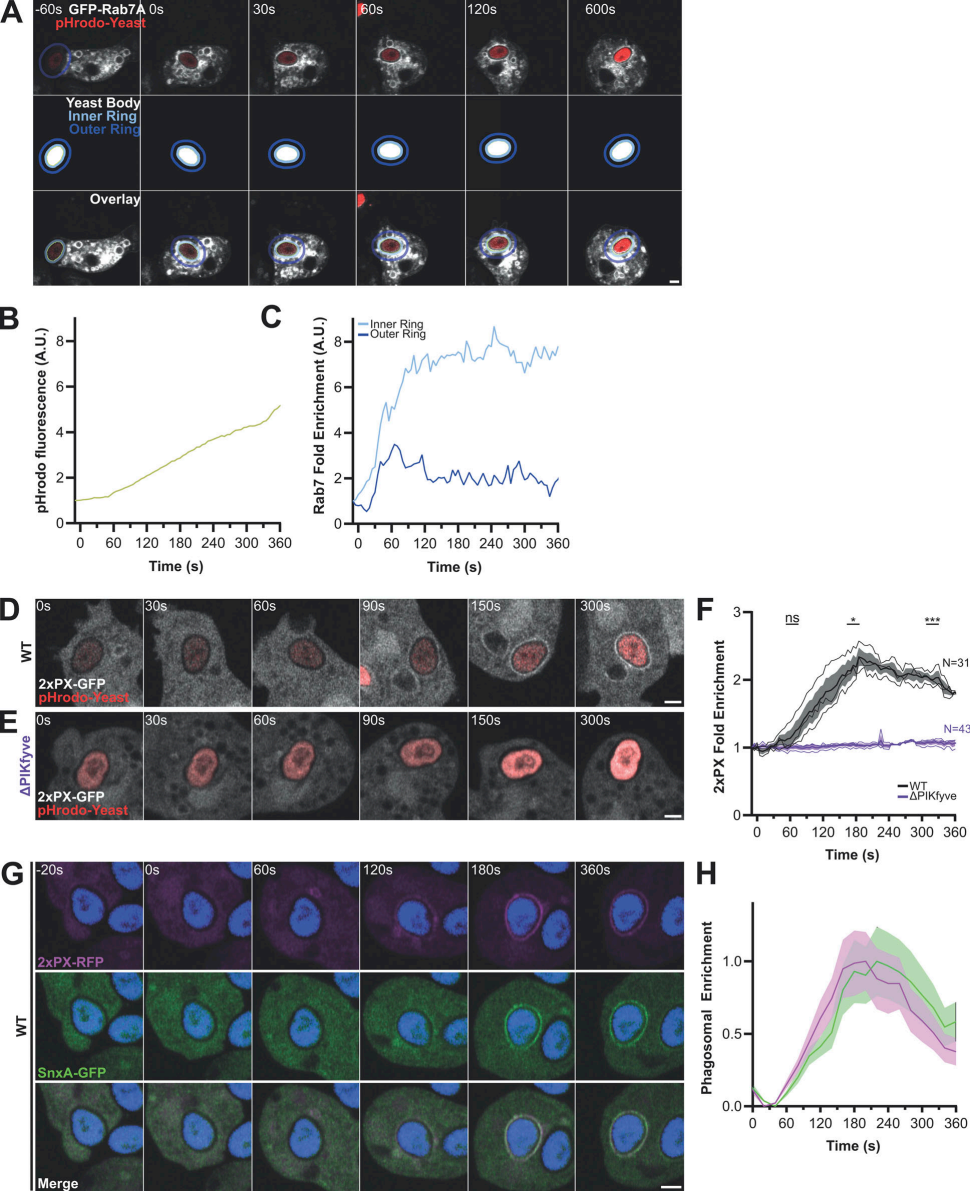
**Quantification of phagosomal recruitment and comparison of SnxA and 2xPX. (A)** Shows automated methodology to quantify fluorescent reporter recruitment to a phagocytic event. Channel containing yeast (red) is thresholded and particles larger than 1.5 μm^2^ are segmented (white circles). **(B)** This area is used to monitor fluorescence change of pHrodo-labeled-yeast, a proxy for phagosomal pH. **(C)** The perimeter is enlarged by 0.2 μm to generate a ring of 0.3 μm thickness over the phagosomal membrane (inner ring). As a control, an additional ring further away from the yeast (outer ring, expanded by 0.85 μm from the yeast) was also generated to give a measure of background cytosol fluorescence that should remain constant. **(D)** Timelapse of 2xPX-GFP recruitment to a pHrodo-labeled yeast containing phagosome in WT cells. **(E)** An identical experiment in ΔPIKfyve mutants. **(F)** Quantification of phagosomal enrichment in both cell lines. N indicates the total number of phagosomes measured, across three independent experiments. *P < 0.05, ***P < 0.005. Graph shows the mean ± SEM across independent experiments; thin lines show each of the individual repeats. **(G)** Timelapse of phagocytosis in WT cells coexpressing SnxA-GFP and 2xPX-RFP (see Video 3 for full timeseries). **(H)** Quantification of probe intensities over time in this video.

We then observed SnxA-GFP recruitment to phagosomes using timelapse confocal microscopy of cells engulfing pHrodo-labeled yeast. Unlike macropinosomes, individual yeast containing phagosomes could be reliably followed over time, so we also developed an automated image analysis pipeline to measure the fluorescence intensity in a ring around the yeast and average over multiple events (Fig. S4, A–C). This showed that phagosomes also acquire SnxA-GFP 2 min after engulfment, with no enrichment observed in ΔPIKfyve controls (Fig. 4, C–E; and Video 2). In contrast to the rapid loss from macropinosomes, longer-term time lapses indicated that SnxA-GFP then remained on the phagosomal membrane for a further 20 min (Fig. 4, F and G). Therefore, although the early stages of macropinosome and phagosome maturation appear identical, the timing then appears to diverge.

**Video 2.** **PI(3,5)P2 dynamics during phagosome maturation.** WT (left) and ΔPIKfyve (right) cells expressing the SnxA-GFP probe for PI(3,5)P2 (white). Red particles are pHrodo-labeled yeast. Note the complete loss of localization in the mutants. Relates to Fig. 4, C and D. Images were captured every 5 s by spinning-disc microscopy and displayed at seven frames per second. Scale bar = 2 μm.

The quantitative analysis of phagosome maturation also allowed us to compare recruitment of 2xPX-GFP to full-length SnxA-GFP. This showed that 2xPX-GFP was recruited to phagosomes with identical dynamics to the full-length SnxA, but whereas SnxA-GFP remained stably associated with the phagosome (Fig. 4 E), the intensity of 2xPX-GFP slowly decreased after 3 min (Fig. S4, D–F). Co-expression of 2xPX-RFP in cells expressing SnxA-GFP showed that this effect was dominant as both reporters were then lost with comparable dynamics (Fig. S4, G and H; and Video 3). Therefore, 2xPX expression again exhibited a greater tendency to interfere with PI(3,5)P_2_ signaling than SnxA-GFP, consistent with our observations in mammalian cells. However, in *Dictyostelium* at least, this was relatively minor and insufficient to detectably perturb phagosomal digestion. As 2xPX had superior contrast to full-length SnxA as an RFP fusion, with indistinguishable initial recruitment, it is therefore still useful in some circumstances, with appropriate caution.

**Video 3.** **Colocalization of SnxA-GFP (green) and 2xPX-RFP (magenta) to a newly formed phagosome containing an Alexa405-labeled yeast (blue) in a WT** ***Dictyostelium* cell.** Images were captured every 20 s by spinning-disc microscopy and displayed at a rate of seven frames per second. Scale bar = 2 μm.

### The SnxA reporter reveals complex coordination of PIKfyve recruitment and activity

PI(3,5)P_2_ is generated by phosphorylation of PI(3)P, which is both the substrate of PIKfyve and responsible for its recruitment via the FYVE domain. SnxA allows us to observe for the first time PI(3,5)P_2_ accumulation in live cells, so we next asked how this relates to both PI(3)P and PIKfyve dynamics.

**Figure 5.**
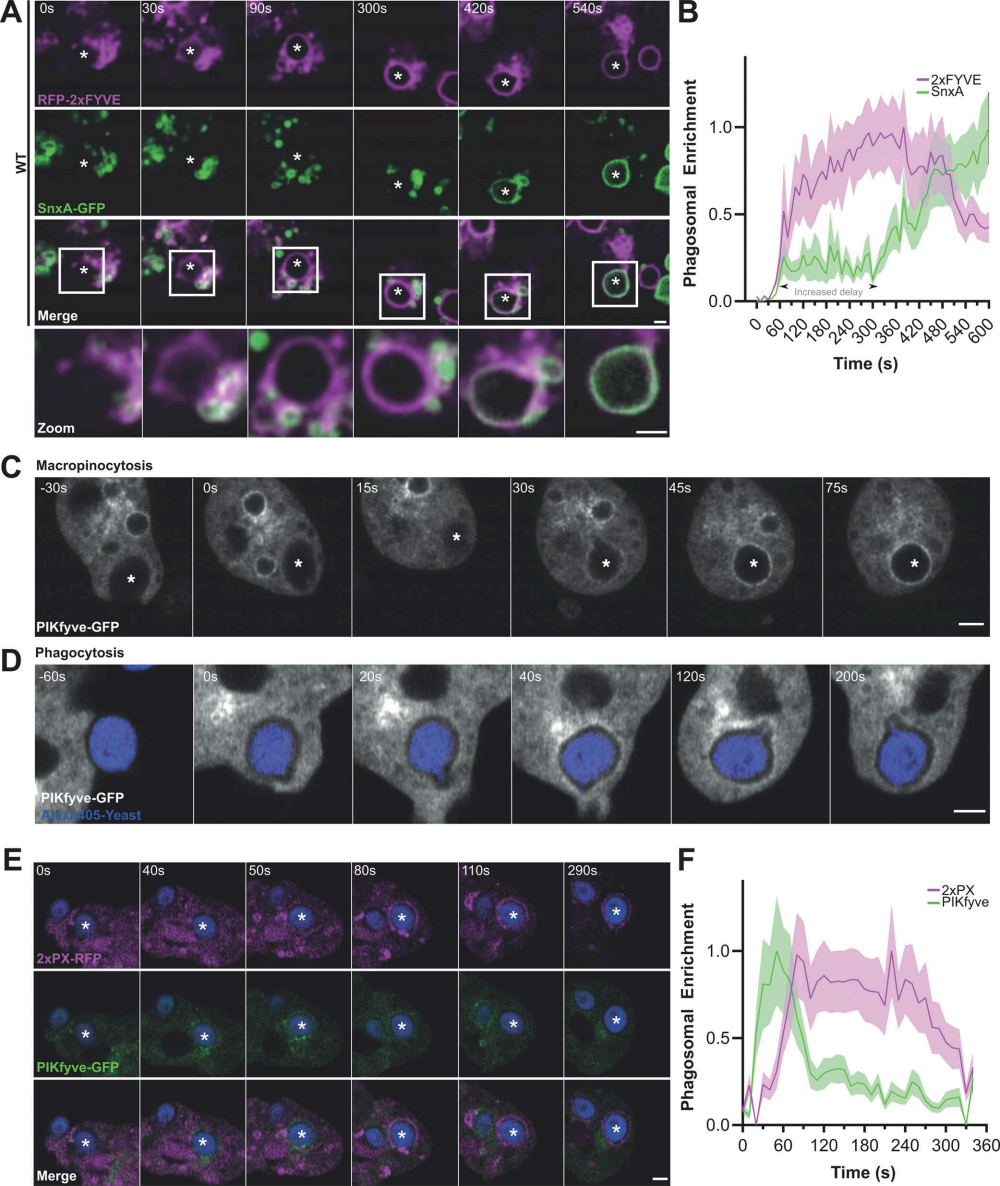
**Recruitment of SnxA relative to PI(3)P and PIKfyve. (A)** Representative timelapse of cells co-expressing 2xFYVE-RFP (magenta) and SnxA-GFP (green) during phagocytosis of an unlabeled yeast. Engulfed yeast is indicated with an asterisk; scale bar = 2 μm. See Video 4 for full-time series. **(B)** Quantification of the fluorescence intensities of both channels around the yeast in this video. **(C and D)** Recruitment of GFP-PIKfyve to a newly formed macropinosome and phagosome, respectively. The full timelapse is shown in Videos 5 and 6. **(E)** Timelapse of cells co-expressing 2xPX-RFP (magenta) and PIKfyve-GFP (green) during phagocytosis of Alexa405-yeast (blue). **(F)** Quantification of the fluorescence intensities around the phagosome in E. Data shown is the mean intensity ± the SD around the phagosomal membrane normalized between minimum and maximum intensities observed.

To compare the relative dynamics of PI(3)P and PI(3,5)P_2_, we co-expressed SnxA-GFP with the PI(3)P reporter RFP-2xFYVE (Gillooly et al., 2000) and followed early phagosome maturation. Although RFP-2xFYVE expression caused a delay in SnxA-GFP recruitment compared with our previous experiments, it was clearly recruited first, immediately following engulfment (Fig. 5, A and B; and Video 4). SnxA-GFP was then enriched on the phagosome several minutes later (Fig. 5, A and B; and Video 4). In this experiment, this was accompanied by a partial drop in RFP-2xFYVE intensity; this is consistent with PIKfyve depleting PI(3)P as indicated in mammalian cells (Hazeki et al., 2012; Kim et al., 2014), although our previous studies show that PI(3)P dynamics are unaffected by loss of PIKfyve in *Dictyostelium* (Buckley et al., 2019). Either way, our observations confirm the early formation of PI(3)P and subsequent conversion to PI(3,5)P_2_ by phagosomal PIKfyve (Ellson et al., 2001; Hazeki et al., 2012).

**Video 4.** **Simultaneous imaging of PI(3)P (RFP-2xFYVE, magenta) and PI(3,5)P2 (SnxA-GFP, green) recruitment to a yeast-containing phagosome (unlabeled) in WT cells.** Images were captured every 10 s by spinning-disc microscopy and displayed at five frames per second. The yeast is annotated by an asterisk. Scale bar = 2 μm.

**Figure S5.**
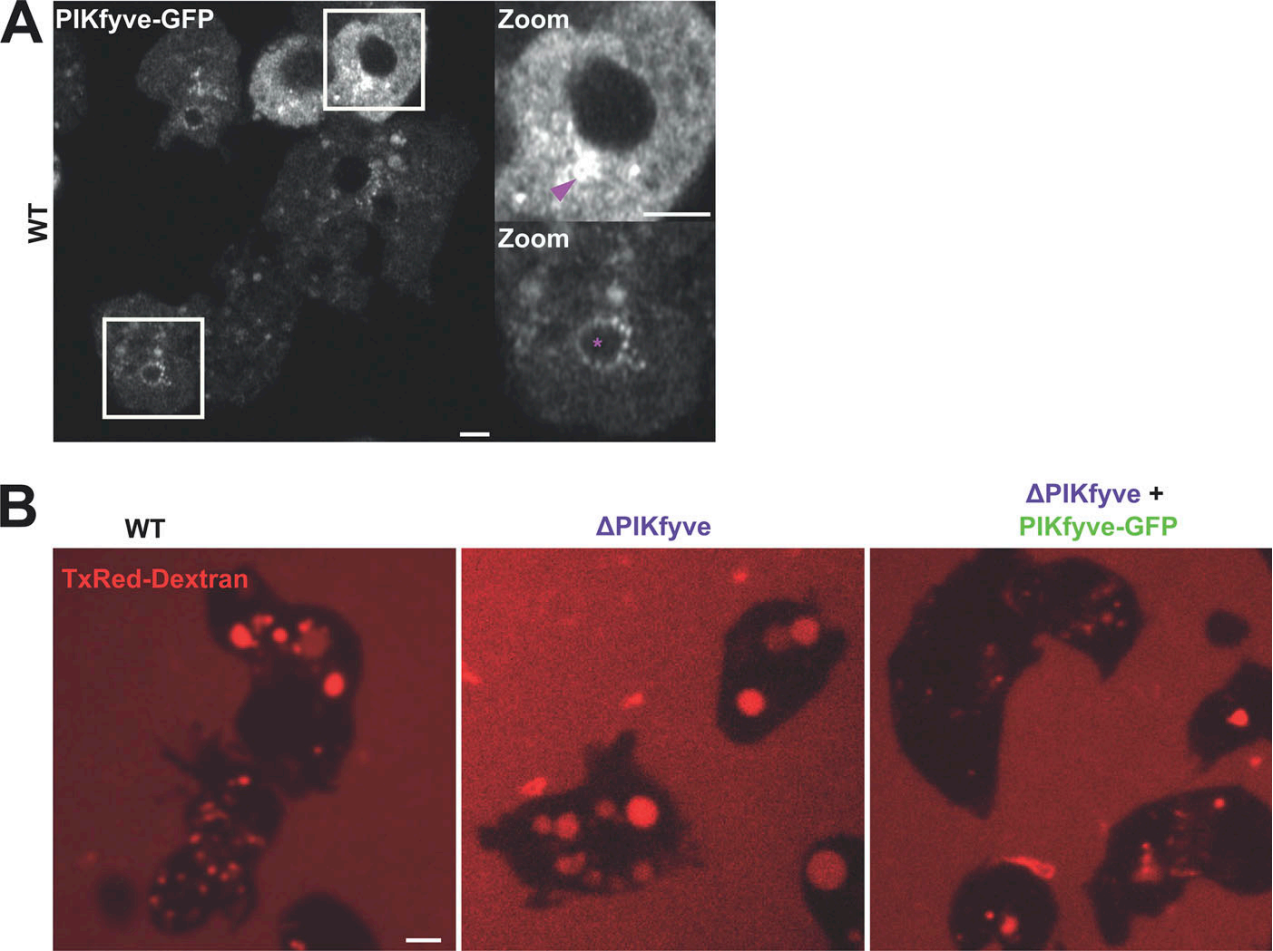
**Expression of PIKfyve-GFP in *Dictyostelium.* (A)** Localization of PIKfyve-GFP in WT cells. Boxes indicate regions enlarged in the panels on the right; arrowhead indicates perinuclear cluster of vesicles. **(B)** Rescue of the macropinosome concentration defect by PIKfyve-GFP expression in ΔPIKfyve cells. The cell lines indicted were incubated overnight in Texas-Red dextran overnight prior to confocal microscopy. Scale bar = 2 μm.

We then investigated how the dynamics of PIKfyve related to PI(3,5)P_2_ accumulation by expression of PIKfyve-GFP. Although PIKfyve-GFP expressed poorly in WT cells, it was clearly visible on large vesicles that resembled macropinosomes (Fig. S5 A). A diffuse perinuclear cluster of presumably subresolution vesicles could also be observed in some cells. Contrast was improved by expression in ΔPIKfyve cells due to loss of competition with the endogenous protein, which also rescued the swollen endosomal phenotype (Fig. S5 B). This allowed PIKfyve-GFP dynamics to be observed over time, demonstrating recruitment to both macropinosomes and phagosomes within 60 s of engulfment (Fig. S5, C and D; and Videos 5 and 6). This is consistent with the role of PIKfyve in the early maturation and its initial recruitment by PI(3)P. Although low signal and movement in the Z-axis meant it was not possible to reliably follow individual macropinosomes much beyond ∼2 min, we observed removal of PIKfyve-GFP from phagosomes ∼3 min after engulfment.

**Video 5.** **Recruitment of PIKfyve-GFP to a newly formed macropinosome (asterisk).** PIKfyve-GFP expressed in a ΔPIKfyve cell. Images were captured every 5 s by spinning-disc microscopy and displayed at five frames per second. Scale bar = 2 μm.

**Video 6.** **Recruitment of GFP-PIKfyve to phagosomes.** Engulfment of an Alexa405-labeled yeast (blue) by a GFP-PIKfyve (white) expressing ΔPIKfyve cell. Images were captured every 20 s by spinning-disc microscopy and displayed at five frames per second. Scale bar = 2 μm.

To examine how PIKfyve recruitment and dissociation relate to PI(3,5)P_2_ production, we coexpressed PIKfyve-GFP with the SnxA probe (RFP-2xPX) in cells engulfing Alexa-405–labeled yeast. This confirmed that PIKfyve is recruited 1–2 min prior to PI(3,5)P_2_ accumulation but also showed that they only colocalize fleetingly, with PIKfyve-GFP dissociating immediately after the SnxA probe arrives (Fig. 5, E and F; and Video 7). PIKfyve is therefore recruited early during phagosome maturation but dissociates rapidly upon activation and accumulation of PI(3,5)P_2_.

**Video 7.** **Simultaneous imaging of PIKfyve-GFP (green) and PI(3,5)P2 formation (RFP-2xPX, in magenta) in WT cells engulfing an Alexa405-labeled yeast (blue).** Images were captured every 10 s by spinning-disc microscopy and displayed at five frames per second. Asterisk indicates the engulfed yeast. Scale bar = 2 μm.

**Figure 6.**
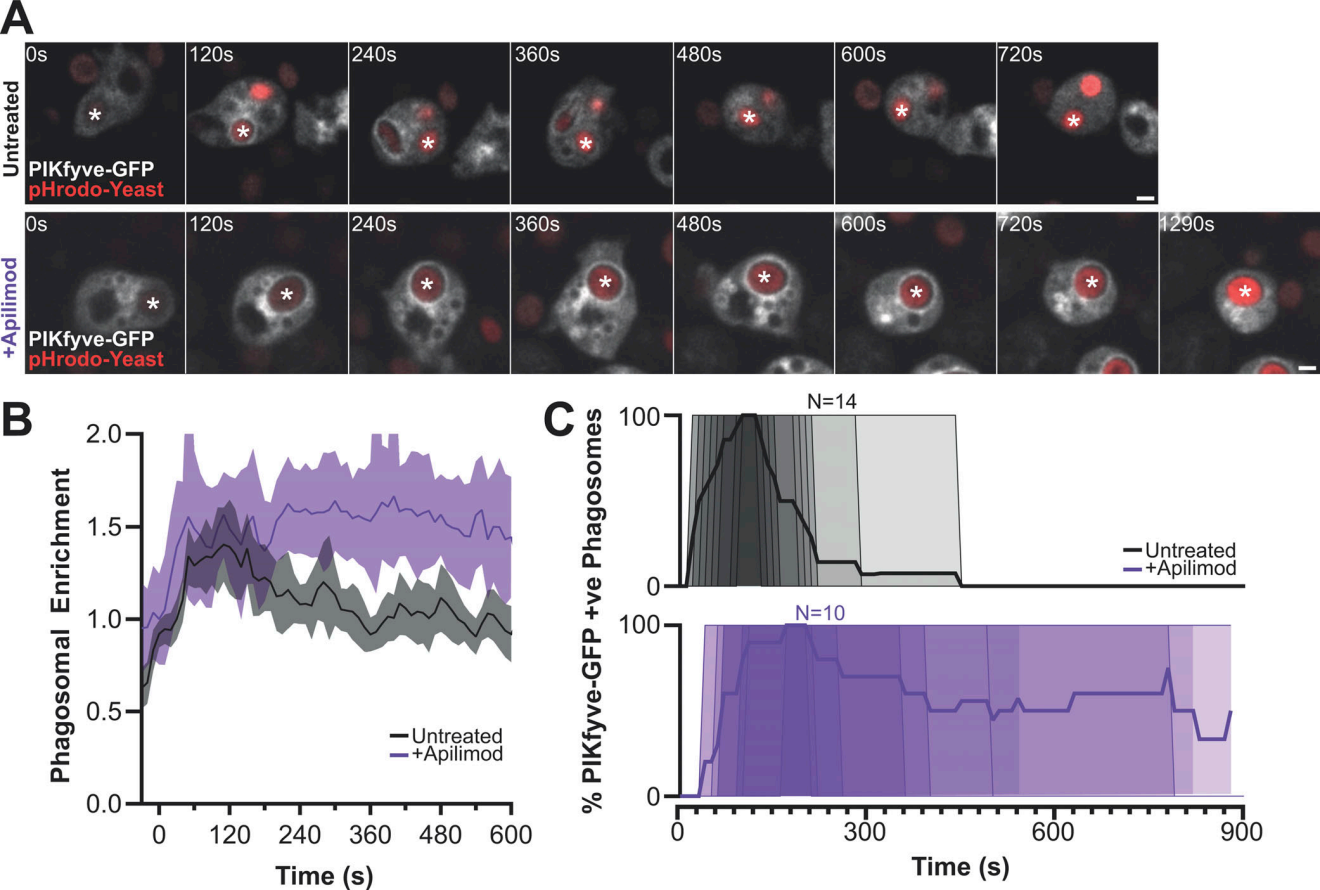
**PIKfyve activity is required for its dissociation from phagosomes. (A)** Timelapses of PIKfyve-GFP expressing cells during phagocytosis of pHrodo-yeast (red) before (top) and after (bottom) addition of 5 μM apilimod. **(B)** The PIKfyve-GFP intensity around the phagosome in these movies. **(C)** PIKfyve-GFP dynamics over multiple events by manually scoring the time of PIKfyve-GFP delivery and release. 14 untreated events and 10 apilimod-treated events were scored over three experiments, each with the thick line indicating the proportion of GFP-positive phagosomes at each time after engulfment. Thin lines represent individual events. All scale bars = 2 μm.

As PI(3)P is present throughout the first 10 min of both macropinosome and phagosome maturation (Buckley et al., 2019; King and Kay, 2019), dissociation of PIKfyve at 2–3 min indicates an additional layer of regulation. As PIKfyve dissociation coincides with PI(3,5)P_2_ accumulation, we used the inhibitor apilimod to test whether PIKfyve activity is required for its release. Consistent with this, PIKfyve-GFP appeared to accumulate more strongly on phagosomes in aplimod-treated cells. Timelapse microscopy then confirmed that apilmod treatment caused PIKfyve to be retained on phagosomes for as long as we could follow them (∼15 min), compared to 4 min in untreated controls (Fig. 6, A–C). This demonstrates that PIKfyve dissociation requires its catalytic activity, providing a mechanism to couple activation and release.

## Discussion

In this study, we identify the *Dictyostelium* protein SnxA as a highly selective PI(3,5)P_2_-binding protein and demonstrate its use as a reporter for PIKfyve signaling in both *Dictyostelium* and mammalian cells. Using SnxA-GFP fusions, we have been able to visualize PI(3,5)P_2_ dynamics in live cells for the first time and provide insight into the complex regulation of PIKfyve and the early regulation of phagosome and macropinosome maturation.

Whilst we find that both full-length SnxA and tandem PX domain constructs are selective for PI(3,5)P_2_, we note several caveats that should be considered with their use. First, the full-length protein contains a coiled-coil domain in addition to the PI(3,5)P_2_-binding PX domain. The primary function of this appears to be mediating homodimerization, which is highly advantageous in increasing binding avidity, but we cannot completely exclude interactions with other proteins. Nonetheless, with the exception of the moderate PI(3,5)P_2_-independent enrichment at the plasma membrane observed upon paraformaldehyde fixation, full-length SnxA is recruited to the same compartments with comparable dynamics to a 2xPX construct lacking the coiled-coil domain. Therefore, any additional interactions appear to have minor or no effects on localization.

The 2xPX construct is theoretically superior as it has the same K_d_ as a SnxA dimer and should be more specific. However, we observed tandem PX domain fusions also have a greater tendency to disrupt PI(3,5)P_2_ signaling in both *Dictyostelium* and mammalian cells. Though disruption was minor in *Dictyostelium*, cell morphology, growth, and macropinocytosis were clearly perturbed in some mammalian cell lines. This is most likely due to the 2xPX probe outcompeting endogenous PI(3,5)P_2_ effectors or regulators. We speculate this is due to a slower off-rate of a constitutive tandem fusion compared with a SnxA dimer where the domains are more spaced and connected by transient electrostatic interactions.

Despite this caveat, the 2xPX probe often had better contrast for microscopy. This was especially evident when expression levels varied over a large range or when imaging parameters were challenging, such as in co-expression experiments. In *Dictyostelium* cells expressing SnxA-GFP from an extrachromosomal vector, vesicular localization was only visible above the background signal in lower expressing cells, whereas 2xPX-GFP and particularly 2xPX-RFP performed much better than their counterparts. Deleterious effects of 2xPX expression in mammalian cells were also not evident in all cell lines tested and are likely to be minimized by low expression, using weaker promoters than those used in this study. It is also possible to bypass probe expression artifacts altogether by using recombinant probes to stain fixed cells (Hammond et al., 2009; Maib and Murray, 2022). The choice of full-length SnxA or 2xPX probes, therefore, depends on individual experimental conditions. We recommend future users carefully test both using appropriate controls including treatment with PIKfyve inhibitors and functional readouts of the pathways under investigation where possible.

The ability to observe the localization and dynamics of both the PIKfyve enzyme and its product has provided several new mechanistic insights. Combined, our data support a model where PIKfyve is regulated in a series of coordinated steps: Initially, inactive PIKfyve is recruited by binding PI(3)P via its FYVE domain before subsequent activation causes rapid PI(3,5)P_2_ accumulation. Finally, PIKfyve dissociates depending on its own activity.

Complex regulation is not unexpected. PIKfyve is unusual in that its catalytic substrate (PI(3)P) also mediates its recruitment via additional interactions with the FYVE domain. While PIKfyve could be released from the membrane by simply using up all the PI(3)P (Kerr et al., 2010), this is unlikely to be the case in *Dictyostelium* as significant PI(3)P is retained on phagosomes long after PIKfyve dissociates and loss of PIKfyve does not affect PI(3)P dynamics (Buckley et al., 2019). There is therefore likely to be additional regulation of the FYVE domain binding. This is supported by a recent study demonstrating recruitment of the yeast PIKfyve ortholog (Fab1) is regulated by phosphorylation near its FYVE domain by TORC1, causing translocation between the yeast vacuole and signaling endosomes (Chen et al., 2021). Although it is unclear whether this mechanism is conserved in other endocytic pathways or organisms, the finding that PIKfyve recruitment to PI(3)P is also conditionally regulated during *Dictyostelium* phagosome maturation highlights strong similarities.

A further complication is that PIKfyve is complexed with the phosphatase Fig4, which can potentially break down PI(3,5)P_2_ as soon as it is generated. As PI(3,5)P_2_ accumulates in a burst almost 2 min after PIKfyve arrival, either PIKfyve or Fig4 catalytic activity must also be regulated. The partial structure of the PIKfyve complex suggests that PIKfyve can be inhibited by autophosphorylation, requiring protein dephosphorylation activity of Fig4 for activation (Lees et al., 2020). Fig4 may therefore also be temporally regulated during phagosome maturation or an additional unknown regulatory mechanism may exist. Our observations that PIKfyve dissociates as soon as PI(3,5)P_2_ accumulates but is retained if pharmacologically inactivated indicates that catalytic activity also affects membrane association. This is consistent with a previous study in mammalian cells, where catalytically inactive PIKfyve was also retained on macropinosomes (Kerr et al., 2010). Although the FYVE domain is not well resolved in the published structure, it is suggested to lie near the kinase domain (Lees et al., 2020). This may provide potential for an additional level of regulation to explain the coordination of recruitment and activity in vivo but needs more investigation.

Many aspects of maturation are shared between phagosomes, macropinosomes, and other endocytic pathways, such as classical clathrin-mediated endosomes. These also transition from a Rab5/PI(3)P-positive early form to later compartments demarked by Rab7, vacuolar ATPase, and lysosomal components. Furthermore, degradation of classically endocytosed receptors, autophagosomes, and entotic vesicles are also sensitive to PIKfyve inhibition (de Lartigue et al., 2009; Krishna et al., 2016; Qiao et al., 2021). A significant limitation of SnxA as a PI(3,5)P_2_ probe is that we were not able to see any clear recruitment to these smaller endocytic compartments. There are several potential explanations for this. For example, PI(3,5)P_2_ may not be present in sufficient abundance in other endocytic pathways due to their limited membrane surface, or a limited pool of PI(3,5)P_2_ may already be fully occupied by endogenous binding proteins. Low levels of SnxA recruitment onto small compartments may also simply not be visible over the cytosolic background with standard confocal imaging.

Whatever the reason, it is likely that our observations with phagosomes and macropinosomes are applicable to other endocytic routes and overcome the technical challenges of following ∼100 nm vesicles in sufficient detail over time. Using *Dictyostelium* SnxA to report PI(3,5)P_2_ dynamics in live cells, we have harnessed an experimentally tractable approach to define the complex sequence of events that occur in the first minutes of phagosome maturation with key mechanistic insights into regulation of PIKfyve and PI(3,5)P_2_.

## Materials and methods

### *Dictyostelium* culture

All *D. discoideum* cells were derived from the Ax2 (Kay) laboratory strain background (strain ID: DBS0235521) and grown in adherent culture in filter-sterilized HL5 medium (Formedium) at 22°C. Cells expressing extrachromosomal plasmids were transformed by electroporation and grown in appropriate antibiotic selection by addition of either 20 µg/ml hygromycin (Invitrogen) or 10 µg/ml G418 (Sigma-Aldrich). The PIKfyve knockout strain in Ax2 background was generated as previously described (Buckley et al., 2019). Live cell imaging was performed in defined SIH medium (Formedium).

Growth on bacterial lawns was performed by seeding ∼20 *Dictyostelium* cells with suspension of *Klebsiella aerogenes* on Sussman's Media agar plates (Formedium). Colonies were visible after 5, and colony size was measured each day. Tests of growth on a panel of bacteria were performed as described by Froquet et al. (2009). Dilutions from 1 to 1 × 10^4^ cells were seeded on agarose in wells of a 96-well plate containing the relevant bacteria. After several days (dependent on bacterial strain), wells were scored for the presence of *Dictyostelium* colonies. Bacteria used were a gift from Pierre Cosson (University of Geneva, Geneva, Switzerland) and were *Klebsiella pneumoniae* laboratory strain and 52145 isogenic mutant (Benghezal et al., 2006), *Micrococcus luteus* (Wilczynska and Fisher, 1994), the isogenic *Pseudomonas aeruginosa* strains PT5 and PT531 (*rhlR-lasR* avirulent mutant; Cosson et al., 2002), *Escherichia coli* B/r (Gerisch, 1959), non-sporulating *Bacillus subtilis* 36.1 (Ratner and Newell, 1978), and *E*. *coli* DH5α (Thermo Fisher Scientific). An avirulent strain of *K*. *pneumoniae* was obtained from ATCC (Strain no. 51697).

### Mammalian cell culture

HEK293, Mia-PaCa-2, and RPE-1 cells were cultured in DMEM supplemented with 10% FBS, 2 mM L-glutamine, and 50 μg/ml penicillin/streptomycin at 37°C in a humidified incubator with 5% CO_2_. For transient transfection with SnxA-GFP or 2xPX-GFP probes, cells were seeded in glass-bottomed dishes or coverslips at 200,000 cells/ml and grown overnight before treatment with Lipofectamine 3000 Transfection Reagent as per manufactures guidelines. For apilimod treatment, 1 μM apilimod was added 24 h after transfection, for at least 1 h prior to imaging.

To score the number of GFP-positive vesicles in live cells, 10 images were captured per condition per repeat. Cells were scored for the number of identifiable vesicles within the cell. A Z-stack was used to help identify vesicles, although single Z-stacks are shown in figures for clarity. Untransfected cells or those with extremely high levels of expression were not counted. To see dextran uptake in live cells, media containing 2.5 mg/ml 70 kD Texas-Red dextran (Life Technologies) was added. Images were captured every 60 s for up to an hour. For fixation, cells on coverslips were first washed twice in PBS and treated with 4% PFA for 10 min before another wash and mounting. All mammalian cell images were captured using a Nikon W1 Spinning Disk confocal using a Plan Apo λ 100×1.4NA oil objective using a Prime 95B A19F203018 camera. Acquisition was controlled via Nikon NIS elements. Live cells were imaged at 37°C and 5% CO_2_ in their DMEM growth medium.

## Cloning

All gene sequences used for cloning were obtained from dictybase (http://www.dictybase.org/; Fey et al., 2013). PX-domain-containing proteins encoded within the *Dictyostelium* genome were identified by Basic Local Alignment Search Tool. Full-length SnxA-GFP (pJSK619) and PIKfyve-GFP (pJV0025) plasmids were generated by PCR of the respective coding sequences from cDNA using primeSTAR max DNA polymerase (Clontech), subcloning into Zero Blunt TOPO II vector (Life Technologies), and then cloning into the BglII/SpeI sites of the pDM1045 expression vector (Paschke et al., 2018; Veltman et al., 2009). To generate 1x and 2xPX expression plasmids, the sequence for the PX domain was synthesized by DC Biosciences with a 5′BglII sites and a 5′BamHI and SpeI sites. This was cloned into the Bglll/SpeI sites of pDM1043 to give the 1xPX-GFP expression vector pJV0010. A second copy of the PX domain was then inserted into the BglII sites of this as a BglII/BamHI fragment of the synthesized gene to give the 2xPX-GFP construct pJV0016. The C-terminal mCherry fusion expression vector pDM1097 was used to generate the 2xPX-RFP expression vector. The same procedure was used to generate the mammalian expression vectors. The full-length and PX-domain-coding sequences were human codon optimized, synthesized, and cloned as BamHI/EcoRI fragments into pEGFP-C1. The full-length, 1xPX, and 2xPX constructs are pJSK659, 663, and 664, respectively. These will be available at http://Addgene.com.

2xFYVE-GFP (pJSK418) uses the sequence from the human Hrs gene and was previously described (Calvo-Garrido et al., 2014). Co-expression plasmids were created by first cloning the RFP-fusion sequence into the pDM series shuttle vectors (pDM1042 and pDM1121) before cloning as an NgoMIV fragment into the appropriate GFP-expression vector. The SnxA-GFP integrating vector was made by cloning full-length SnxA into the integrating C-terminal GFP vector pDM1053 (Paschke et al., 2018). Prior to transformation, the plasmid was linearized with BamHI and transformation was performed with 100 U of BamHI, producing a 5–10-fold increase in the number of colonies compared with controls. Colonies were selected and screened by confocal microscopy for a cell line with low expression and high contrast.

### PIP arrays

PIP arrays were performed using whole cell lysates from *Dictyostelium* cells expressing the GFP-fusion constructs. Approximately, 2 × 10^8^ cells were lysed in radioimmunoprecipitation assay buffer (50 mM Tris HCL, pH 7.5, 150 mM NaCl, 0.1% SDS, 2 mM EDTA, 0.5% sodium deoxycholate, 1× Halt protease inhibitor cocktail [Pierce]). PIP arrays (Echelon Biosciences) were blocked for 1 h at room temperature in 3% fatty-acid-free BSA in Tris-buffered saline with 0.1% tween and then incubated with lysates for 1 h. Membranes were incubated with a custom rabbit polyclonal anti-GFP antibody (gift from Andrew Peden, University of Sheffield, Sheffield, UK) for 1 h at room temperature before washing and then Alexa Fluor 800–conjugated anti-rabbit secondary antibody (A32735; Life Technologies) was added before imaging in a LiCor Odyssey SA fluorescent imager.

### Purification of recombinant proteins

All SnxA probes were expressed as N-terminal 6xHis and eGFP fusion proteins in BL21 cells in Lysogeny Broth media containing ampicillin and 1.7 g lactose per liter for 7 h at 37°C before decreasing to 18°C for ∼10–12 h. Cells were pelleted and lysed by ultrasound before clearing by centrifugation at 60,000 × *g* for 1 h. Cleared lysates were filtered through a 0.22-µm filter and purified using a 5 ml His trap HP column followed by anion exchange using a 5 ml Capto-Q column and size exclusion chromatography using a Superdex 200 16/60 pg column. All probes were snap-frozen in liquid nitrogen and stored at −80°C.

### Lipid-binding experiments

The experiments were performed as previously described (Maib and Murray, 2022). 1 mg liposomes, containing each of the seven different phosphatidylinositols were produced by mixing 95 mol % 1-palmitoyl-2-oleoyl-sn-glycero-3-phosphocholine with 5 mol % of the respective phosphatidylinositol together with 0.1% Atto647N-DOPE. The mixtures were evaporated under nitrogen and dried overnight in a vacuum extruder. Dried lipids were resuspended in 1 ml buffer containing 150 mM NaCl, 20 mM Hepes, and 0.5 mM tris(2-carboxyethyl)phosphine hydrochloride (TCEP) and subjected to six freeze–thaws in liquid nitrogen. Liposomes ∼100 nm diameter were generated by passing the lipid mixture 11 times through a 100-nm filter (Whatman Nuclepore). Liposomes were aliquoted, snap-frozen, and stored at −20°C.

Membrane-coated beads were generated by adding 10 µg of liposomes to ∼0.5 × 10^6^ 10 µm silica beads (Whitehouse Scientific) in 100 μl of 200 mM NaCl for 30 min rotation at room temperature. Beads were washed twice and resuspended in a buffer containing 150 mM NaCl, 20 mM Hepes, and 0.5 mM TCEP and blocked with 10% goat serum for 30 min. Drops of 7.5 μl beads were added into the corners of uncoated µ-Slide 8-well chambers (Ibidi) and 7.5 μl of the purified SnxA probes was added and mixed by pipetting at indicated final concentrations. Lipid-binding kinetics were allowed to equilibrate for 30 min at room temperature before imaging close to the equator of the beads.

Confocal images were acquired using a Leica SP8 Confocal Microscope with a Leica HC PL APO CS2 63×/1.40 Oil objective at 0.75 base zoom with 1,024 × 1,024 pixels scan. GFP and Atto647N-DOPE fluorescence were imaged simultaneously without any measurable bleed-through. Data were analyzed using a custom ImageJ script that segments the Atto647N-DOPE channel to create a mask around the outer circumference of each bead. Segmented masks were then used to measure GFP fluorescence around each bead.

### Mass photometry

Mass photometry measurements were performed as described in Sonn-Segev et al. (2020). Briefly, coverslips were cleaned in 50% isopropanol, and 5 μl of sample was applied to a culture well gasket. Samples were diluted to a concentration between 25 and 50 nM in buffer containing 250 mM NaCl, 20 mM Hepes, and 0.5 M TCEP immediately before measurements. Data were acquired using OneMP mass photometer (Refeyn Ltd) AcquireMP (Refeyn Ltd), and data analysis was performed using DiscoverMP (Refeyn Ltd). Calibration was carried out using standards of known molecular weight. For each sample, between 1,315 and 10,380 individual events were recorded and analyzed.

### Preparation of fluorescent yeast and dextran

*Saccharomyces cerevisiae* were used for phagocytosis assays. Yeast were grown for 3 d at 37°C in standard yeast peptone dextrose media (Formedium) until stationary phase before centrifugation at 1,000 × *g* for 5 min, resuspended at a final concentration of 1 × 10^9^ cells/ml in PBS, pH 7, and frozen until use. Yeast were fluorescently labeled using either pHrodo red succinimidyl ester (Life Technologies) or Alexa-405 succinimidyl ester (Life Technologies) at a final concentration of 0.25 and 2.5 mM, respectively. 0.5 × 10^9^ yeast were resuspended in 200 μl of PBS at pH 8.5 and incubated with 10 μl of prepared dye for 30 min at 37°C with gentle shaking. After, cells were pelleted and sequentially washed in 1 ml of PBS, pH 8.5, 1 ml of 25 nM Tris-HCl, pH 8.5, and 1 ml of PBS, pH 8.5, again. Finally, cells were resuspended in 500 μl of KK2, pH 6.1, and kept at −20°C. Yeast were diluted in SIH to a working concentration of 1 × 10^8^ yeast/ml before use. Texas-Red 70 kD Dextran (Life Technologies) was resuspended in water and diluted to a working concentration of 2 mg/ml.

### Microscopy and image analysis

For fluorescence microscopy, ∼1 × 10^6^ *Dictyostelium* cells were seeded in 35-mm glass-bottomed microscopy dishes (Mat tek P35G-1.5-14-C) and left to grow overnight in SIH medium (Formedium). Cells were imaged on a Zeiss LSM880 AiryScan Confocal with 63×1.4NA Plan Apochromat oil objective and processed using the Zeiss Zen microscope software unless otherwise stated. All *Dictyostelium* imaging was performed at room temperature.

For timelapse microscopy of phagocytosis, most of the medium was aspirated immediately before imaging and 20 μl of 1 × 10^8^ labeled yeast added. After 60 s, cells were overlaid with a thin layer of 1% agarose in SIH and excess medium removed. For normal time lapses, images were captured every 10 s for up to 10 min. For extended time lapses, images were captured every 60 s. Automated analysis of the fluorescent intensity around each phagosomal membrane was performed using the Python plugin pyimagej (https://pypi.org/project/pyimagej/). Images where phagosomal uptake could be seen were manually selected. To segment out the phagosome, the yeast channel was thresholded and particles larger than 1.5 μm^2^ selected. Particles on time-adjacent frames were automatically scored for similarity based on position and size, and high-similarity particles were categorized into the same event. Then, throughout each event, the phagosome area was monitored for fluorescence change of pHrodo-labeled yeast as a proxy for phagosomal pH. The selection perimeter was enlarged into two rings of ∼0.35 μm thickness: an inner ring 0.2 μm enlarged, to measure enrichment at the phagosomal membrane, and an outer ring 0.85 μm enlarged, to monitor background cytosol fluorescence. Each event was then manually screened for errors before mean fluorescence intensity was recorded for each frame, normalized to intracellular background, and fold-enrichment from −10 s calculated. Because addition of an agarose overlay appeared to suppress the effects of apilimod, cells treated with 5 µM apilimod (added >2 h before imaging; USBiological) were imaged without overlay through a larger Z range every 10 s using a Nikon W1 Spinning Disk confocal with a Plan Apo λ 100×1.4NA oil objective using a Prime 95B A19F203018 camera. Acquisition was controlled via Nikon NIS elements. The automated analysis pipeline was not sensitive enough to detect PIKfyve-GFP enrichment, so events were manually followed and scored as either GFP-positive or -negative using ImageJ.

Dextran pulse-chases were performed by aspirating most of the medium and then adding 50 μl of 2 mg/ml 70 kD Texas-Red dextran (11580226; Life Technologies) for the indicated time. Dextran was then removed by washing thrice in SIH medium, leaving ∼1 ml of SIH in the dish. Cells were immediately imaged, with multiple fields of view taken every 2 min for 10 min. GFP colocalization at each time point was then scored manually.

### Generating SnxA knockouts

The SnxA (DDB_G0289833) locus was disrupted by homologous recombination, inserting a blasticidin resistance cassette within the gene and deleting 550 bp of coding sequence. The knockout construct was made using a one-step recombination method into the pKOSG-IBA-Dicty vector as previously described (Wiegand et al., 2011). The 5′ recombination arm was generated with the primers 5′-AGC​GCG​TCT​CCA​ATG​ACC​ACC​CAG​GTT​AAA​AAT​AAT​TCC-3′/5′-AGC​GCG​TCT​CCC​TTC​TTC​CTT​TTC​TAA​GAG​AAT​ATA​TTT​GG-3′, and the 3′ arm with 5′-AGC​GCG​TCT​CCG​TTG​CCT​TAA​AAG​AGA​CGA​AGG​T-3′/5′-AGC​GCG​TCT​CCT​CCC​CTG​GCT​TTG​TTT​TTA​TAA​AAC​AG-3′. PCR products were recombined with the vector using StarCombinase (IBA GmbH). The resulting vector was used to transform Ax2 *Dictyostelium*, and the resulting blasticidin-resistant clones were screened for successful gene disruption by PCR using the primers 5′-GCA​CTG​GGA​GTT​CCA​ATA​TCA​ATA​TCA​TC-3′/5′-ATA​ATT​AAT​TCA​ACA​TCT​TGC​AAA​T-3′. Multiple independent clones were isolated and used for functional characterization.

### Endocytosis and fluid-phase proteolysis

Macropinocytosis and exocytosis were measured, as previously described (Maniak, 2001), by incubating 5 × 10^6^ cells/ml in HL5 medium containing 2 mg/ml 70 kD FITC dextran (FD705; Sigma-Aldrich). 500 μl of cells were then removed at each time point and added to 1 ml ice-cold KK2 (0.1 M potassium phosphate, pH 6.1). Cell pellets were then washed once and lysed in 50 mM Na_2_HPO_4_, pH 9.3, 0.2% Triton X-100 before FITC fluorescence was measured in a fluorimeter. Exocytosis was measured in the same way, except cells were preincubated with FITC-dextran for at least 2 h, before washing out and measuring the decrease in fluorescence over time.

### Phagocytosis assays

Bead uptake, proteolysis, and acidification were performed as previously described in detail in Sattler et al. (2013). Briefly, phagocytosis was measured by uptake of 1 μm YG-green beads (Polysciences Inc.). Cells were incubated with beads for the times indicated and fluorescence was measured by flow cytometry. pH and proteolysis were measured using 3 μm silica beads (Kisker Biotech) conjugated to either FITC or BSA, respectively, as well as Alexa594 as a reference dye. Synchronous phagocytosis was caused by centrifuging beads onto confluent cells in a 96-well plate (1,200 RPM for 10 s) before washing and measuring changes in the fluorescence intensities over time. All samples were measured in triplicate.

### Statistics

Statistical analysis was performed using Graphpad Prism 9. Biological replicate numbers and statistical tests used for each experiment are detailed in each figure legend. P values were calculated by unpaired Students *T* tests assuming a normal distribution, although this was not formally tested. P < 0.05 was deemed significant with * indicating P < 0.05, **P < 0.01, and ***P < 0.005 throughout. All images shown are representative of at least three independent experiments.

### Online supplementary material

Fig. S1 shows additional in vitro characterization of the reporter, including confirmation of recombinant protein purification, absence of binding to PI(5)P, and mass photometry analysis of dimerization. Fig. S2 shows non-specific cortical localization of SnxA-GFP in mammalian cells and lack of dominant effects of probe expression in *Dictyostelium* cells. Fig. S3 shows phenotypic analysis of *snxA* knockout cells. Fig. S4 outlines our automated image analysis pipeline, as well as additional quantification of 2xPX-GFP dynamics in *Dictyostelium*. Fig. S5 provides confirmation of GFP-PIKfyve localization in WT cells and its ability to rescue *PIKfyve* mutant phenotypes. Video 1 shows recruitment of GFP-snxA to macropinosomes in mammalian cells. Video 2 shows PI(3,5)P2 dynamics during *Dictyostelium* phagosome maturation, with a direct comparison between SnxA-GFP and 2xPX-RFP in Video 3. Simultaneous imaging of PI(3)P and PI(3,5)P2 probes during phagosome maturation is shown in Video 4. Videos 5 and 6 show examples of PIKfyve-GFP recruitment to a newly formed macropinosome and phagosome, respectively. Video 7 shows simultaneous imaging of PIKfyve-GFP and PI(3,5)P_2_ dynamics during *Dictyostelium* phagosome maturation.

## Supplementary Material

SourceData F1 is the source file for Fig. 1.

SourceData FS1 is the source file for Fig. S1.

## Data Availability

The raw data and code are available from the corresponding author upon reasonable request.

## References

[bib1] Benghezal, M., M.O. Fauvarque, R. Tournebize, R. Froquet, A. Marchetti, E. Bergeret, B. Lardy, G. Klein, P. Sansonetti, S.J. Charette, and P. Cosson. 2006. Specific host genes required for the killing of Klebsiella bacteria by phagocytes. Cell. Microbiol. 8:139–148. 10.1111/j.1462-5822.2005.00607.x 16367873

[bib2] Berwick, D.C., G.C. Dell, G.I. Welsh, K.J. Heesom, I. Hers, L.M. Fletcher, F.T. Cooke, and J.M. Tavaré. 2004. Protein kinase B phosphorylation of PIKfyve regulates the trafficking of GLUT4 vesicles. J. Cell Sci. 117:5985–5993. 10.1242/jcs.01517 15546921

[bib3] Botelho, R.J., J.A. Efe, D. Teis, and S.D. Emr. 2008. Assembly of a Fab1 phosphoinositide kinase signaling complex requires the Fig4 phosphoinositide phosphatase. Mol. Biol. Cell. 19:4273–4286. 10.1091/mbc.e08-04-0405 18653468 PMC2555960

[bib4] Buckley, C.M., N. Gopaldass, C. Bosmani, S.A. Johnston, T. Soldati, R.H. Insall, and J.S. King. 2016. WASH drives early recycling from macropinosomes and phagosomes to maintain surface phagocytic receptors. Proc. Natl. Acad. Sci. USA. 113:E5906–E5915. 10.1073/pnas.1524532113 27647881 PMC5056073

[bib5] Buckley, C.M., V.L. Heath, A. Guého, C. Bosmani, P. Knobloch, P. Sikakana, N. Personnic, S.K. Dove, R.H. Michell, R. Meier, . 2019. PIKfyve/Fab1 is required for efficient V-ATPase and hydrolase delivery to phagosomes, phagosomal killing, and restriction of Legionella infection. PLoS Pathog. 15:e1007551. 10.1371/journal.ppat.1007551 30730983 PMC6382210

[bib6] Cabezas, A., K. Pattni, and H. Stenmark. 2006. Cloning and subcellular localization of a human phosphatidylinositol 3-phosphate 5-kinase, PIKfyve/Fab1. Gene. 371:34–41. 10.1016/j.gene.2005.11.009 16448788

[bib7] Cai, X., Y. Xu, A.K. Cheung, R.C. Tomlinson, A. Alcázar-Román, L. Murphy, A. Billich, B. Zhang, Y. Feng, M. Klumpp, . 2013. PIKfyve, a class III PI kinase, is the target of the small molecular IL-12/IL-23 inhibitor apilimod and a player in Toll-like receptor signaling. Chem. Biol. 20:912–921. 10.1016/j.chembiol.2013.05.010 23890009 PMC4878021

[bib8] Calvo-Garrido, J., J.S. King, S. Muñoz-Braceras, and R. Escalante. 2014. Vmp1 regulates PtdIns3P signaling during autophagosome formation in Dictyostelium discoideum. Traffic. 15:1235–1246. 10.1111/tra.12210 25131297

[bib9] Chen, D., H. Xiao, K. Zhang, B. Wang, Z. Gao, Y. Jian, X. Qi, J. Sun, L. Miao, and C. Yang. 2010. Retromer is required for apoptotic cell clearance by phagocytic receptor recycling. Science. 327:1261–1264. 10.1126/science.1184840 20133524

[bib10] Chen, Z., P.C. Malia, R. Hatakeyama, R. Nicastro, Z. Hu, M.P. Péli-Gulli, J. Gao, T. Nishimura, E. Eskes, C.J. Stefan, . 2021. TORC1 determines Fab1 lipid kinase function at signaling endosomes and vacuoles. Curr. Biol. 31:297–309.e8. 10.1016/j.cub.2020.10.026 33157024

[bib11] Choy, C.H., G. Saffi, M.A. Gray, C. Wallace, R.M. Dayam, Z.A. Ou, G. Lenk, R. Puertollano, S.C. Watkins, and R.J. Botelho. 2018. Lysosome enlargement during inhibition of the lipid kinase PIKfyve proceeds through lysosome coalescence. J. Cell Sci. 131:jcs213587. 10.1242/jcs.213587 29661845 PMC6031331

[bib12] Cosson, P., L. Zulianello, O. Join-Lambert, F. Faurisson, L. Gebbie, M. Benghezal, C. Van Delden, L.K. Curty, and T. Köhler. 2002. Pseudomonas aeruginosa virulence analyzed in a Dictyostelium discoideum host system. J. Bacteriol. 184:3027–3033. 10.1128/JB.184.11.3027-3033.2002 12003944 PMC135065

[bib13] Dayam, R.M., A. Saric, R.E. Shilliday, and R.J. Botelho. 2015. The phosphoinositide-gated lysosomal Ca(2+) channel, TRPML1, is required for phagosome maturation. Traffic. 16:1010–1026. 10.1111/tra.12303 26010303

[bib14] Dayam, R.M., C.X. Sun, C.H. Choy, G. Mancuso, M. Glogauer, and R.J. Botelho. 2017. The lipid kinase PIKfyve coordinates the neutrophil immune response through the activation of the rac GTPase. J. Immunol. 199:2096–2105. 10.4049/jimmunol.1601466 28779020

[bib15] de Lartigue, J., H. Polson, M. Feldman, K. Shokat, S.A. Tooze, S. Urbé, and M.J. Clague. 2009. PIKfyve regulation of endosome-linked pathways. Traffic. 10:883–893. 10.1111/j.1600-0854.2009.00915.x 19582903 PMC2723830

[bib16] Demirsoy, S., S. Martin, S. Motamedi, S. van Veen, T. Holemans, C. Van den Haute, A. Jordanova, V. Baekelandt, P. Vangheluwe, and P. Agostinis. 2017. ATP13A2/PARK9 regulates endo-/lysosomal cargo sorting and proteostasis through a novel PI(3, 5)P2-mediated scaffolding function. Hum Mol Genet. 26:1656–1669. 10.1093/hmg/ddx070 28334751

[bib17] Dong, X.P., D. Shen, X. Wang, T. Dawson, X. Li, Q. Zhang, X. Cheng, Y. Zhang, L.S. Weisman, M. Delling, and H. Xu. 2010. PI(3,5)P(2) controls membrane trafficking by direct activation of mucolipin Ca(2+) release channels in the endolysosome. Nat. Commun. 1:38. 10.1038/ncomms1037 20802798 PMC2928581

[bib18] Dove, S.K., K. Dong, T. Kobayashi, F.K. Williams, and R.H. Michell. 2009. Phosphatidylinositol 3,5-bisphosphate and Fab1p/PIKfyve underPPIn endo-lysosome function. Biochem. J. 419:1–13. 10.1042/BJ20081950 19272020

[bib19] Ellson, C.D., K.E. Anderson, G. Morgan, E.R. Chilvers, P. Lipp, L.R. Stephens, and P.T. Hawkins. 2001. Phosphatidylinositol 3-phosphate is generated in phagosomal membranes. Curr. Biol. 11:1631–1635. 10.1016/S0960-9822(01)00447-X 11676926

[bib20] Ferguson, C.J., G.M. Lenk, and M.H. Meisler. 2009. Defective autophagy in neurons and astrocytes from mice deficient in PI(3,5)P2. Hum. Mol. Genet. 18:4868–4878. 10.1093/hmg/ddp460 19793721 PMC2778378

[bib21] Fey, P., R.J. Dodson, S. Basu, and R.L. Chisholm. 2013. One stop shop for everything Dictyostelium: dictyBase and the dicty stock center in 2012. Methods Mol. Biol. 983:59–92. 10.1007/978-1-62703-302-2_4 23494302 PMC3762881

[bib22] Froquet, R., E. Lelong, A. Marchetti, and P. Cosson. 2009. Dictyostelium discoideum: A model host to measure bacterial virulence. Nat. Protoc. 4:25–30. 10.1038/nprot.2008.212 19131953

[bib23] Gerisch, G. 1959. Ein submerskulturver fahren Fur entwicklungsphysiologische untersuchungen an Dictyostelium discoideum. Naturwissenschaften. 46:654–656. 10.1007/BF00638009

[bib24] Gillooly, D.J., I.C. Morrow, M. Lindsay, R. Gould, N.J. Bryant, J.M. Gaullier, R.G. Parton, and H. Stenmark. 2000. Localization of phosphatidylinositol 3-phosphate in yeast and mammalian cells. EMBO J. 19:4577–4588. 10.1093/emboj/19.17.4577 10970851 PMC302054

[bib25] Hammond, G.R., G. Schiavo, and R.F. Irvine. 2009. Immunocytochemical techniques reveal multiple, distinct cellular pools of PtdIns4P and PtdIns(4,5)P(2). Biochem. J. 422:23–35. 10.1042/BJ20090428 19508231 PMC2722159

[bib26] Hammond, G.R., S. Takasuga, T. Sasaki, and T. Balla. 2015. The ML1Nx2 phosphatidylinositol 3,5-bisphosphate probe shows poor selectivity in cells. PLoS One. 10:e0139957. 10.1371/journal.pone.0139957 26460749 PMC4604148

[bib27] Hazeki, K., K. Nigorikawa, Y. Takaba, T. Segawa, A. Nukuda, A. Masuda, Y. Ishikawa, K. Kubota, S. Takasuga, and O. Hazeki. 2012. Essential roles of PIKfyve and PTEN on phagosomal phosphatidylinositol 3-phosphate dynamics. FEBS Lett. 586:4010–4015. 10.1016/j.febslet.2012.09.043 23068606

[bib28] Ikonomov, O.C., D. Sbrissa, and A. Shisheva. 2001. Mammalian cell morphology and endocytic membrane homeostasis require enzymatically active phosphoinositide 5-kinase PIKfyve. J. Biol. Chem. 276:26141–26147. 10.1074/jbc.M10172220 11285266

[bib29] Ikonomov, O.C., D. Sbrissa, H. Fenner, and A. Shisheva. 2009. PIKfyve-ArPIKfyve-Sac3 core complex: Contact sites and their consequence for Sac3 phosphatase activity and endocytic membrane homeostasis. J. Biol. Chem. 284:35794–35806. 10.1074/jbc.M109.037515 19840946 PMC2791009

[bib30] Isobe, Y., K. Nigorikawa, G. Tsurumi, S. Takemasu, S. Takasuga, S. Kofuji, and K. Hazeki. 2019. PIKfyve accelerates phagosome acidification through activation of TRPML1 while arrests aberrant vacuolation independent of the Ca2+ channel. J. Biochem. 165:75–84. 10.1093/jb/mvy084 30295876

[bib31] Kerr, M.C., J.T. Wang, N.A. Castro, N.A. Hamilton, L. Town, D.L. Brown, F.A. Meunier, N.F. Brown, J.L. Stow, and R.D. Teasdale. 2010. Inhibition of the PtdIns(5) kinase PIKfyve disrupts intracellular replication of Salmonella. EMBO J. 29:1331–1347. 10.1038/emboj.2010.28 20300065 PMC2868569

[bib32] Kim, G.H., R.M. Dayam, A. Prashar, M. Terebiznik, and R.J. Botelho. 2014. PIKfyve inhibition interferes with phagosome and endosome maturation in macrophages. Traffic. 15:1143–1163. 10.1111/tra.12199 25041080

[bib33] King, J.S., and R.R. Kay. 2019. The origins and evolution of macropinocytosis. Philos. Trans. R. Soc. Lond. B Biol. Sci. 374:20180158. 10.1098/rstb.2018.0158 30967007 PMC6304743

[bib34] Krishna, S., W. Palm, Y. Lee, W. Yang, U. Bandyopadhyay, H. Xu, O. Florey, C.B. Thompson, and M. Overholtzer. 2016. PIKfyve regulates vacuole maturation and nutrient recovery following engulfment. Dev. Cell. 38:536–547. 10.1016/j.devcel.2016.08.001 27623384 PMC5046836

[bib35] Lees, J.A., P. Li, N. Kumar, L.S. Weisman, and K.M. Reinisch. 2020. Insights into lysosomal PI(3,5)P_2_ homeostasis from a structural-biochemical analysis of the PIKfyve lipid kinase complex. Mol. Cell. 80:736–743.e4. 10.1016/j.molcel.2020.10.003 33098764 PMC7962480

[bib36] Leray, X., J.K. Hilton, K. Nwangwu, A. Becerril, V. Mikusevic, G. Fitzgerald, A. Amin, M.R. Weston, and J.A. Mindell. 2022. Tonic inhibition of the chloride/proton antiporter ClC-7 by PI(3,5)P2 is crucial for lysosomal pH maintenance. Elife. 11:e74136. 10.7554/eLife.74136 35670560 PMC9242644

[bib37] Levin, R., S. Grinstein, and J. Canton. 2016. The life cycle of phagosomes: Formation, maturation, and resolution. Immunol. Rev. 273:156–179. 10.1111/imr.12439 27558334

[bib38] Li, S.C., T.T. Diakov, T. Xu, M. Tarsio, W. Zhu, S. Couoh-Cardel, L.S. Weisman, and P.M. Kane. 2014. The signaling lipid PI(3,5)P₂ stabilizes V₁-V(o) sector interactions and activates the V-ATPase. Mol. Biol. Cell. 25:1251–1262. 10.1091/mbc.e13-10-0563 24523285 PMC3982991

[bib39] Li, X., N. Rydzewski, A. Hider, X. Zhang, J. Yang, W. Wang, Q. Gao, X. Cheng, and H. Xu. 2016. A molecular mechanism to regulate lysosome motility for lysosome positioning and tubulation. Nat. Cell Biol. 18:404–417. 10.1038/ncb3324 26950892 PMC4871318

[bib40] Li, X., X. Wang, X. Zhang, M. Zhao, W.L. Tsang, Y. Zhang, R.G. Yau, L.S. Weisman, and H. Xu. 2013. Genetically encoded fluorescent probe to visualize intracellular phosphatidylinositol 3,5-bisphosphate localization and dynamics. Proc. Natl. Acad. Sci. USA. 110:21165–21170. 10.1073/pnas.1311864110 24324172 PMC3876232

[bib41] Maib, H., and D.H. Murray. 2022. A mechanism for exocyst-mediated tethering via Arf6 and PIP5K1C-driven phosphoinositide conversion. Curr. Biol. 32:2821–2833.e6. 10.1016/j.cub.2022.04.089 35609603 PMC9382030

[bib42] Maniak, M. 2001. Fluid-phase uptake and transit in axenic Dictyostelium cells. Biochim. Biophys. Acta. 1525:197–204. 10.1016/S0304-4165(01)00105-2 11257433

[bib43] McCartney, A.J., Y. Zhang, and L.S. Weisman. 2014a. Phosphatidylinositol 3,5-bisphosphate: Low abundance, high significance. BioEssays. 36:52–64. 10.1002/bies.201300012 24323921 PMC3906640

[bib44] McCartney, A.J., S.N. Zolov, E.J. Kauffman, Y. Zhang, B.S. Strunk, L.S. Weisman, and M.A. Sutton. 2014b. Activity-dependent PI(3,5)P2 synthesis controls AMPA receptor trafficking during synaptic depression. Proc. Natl. Acad. Sci. USA. 111:E4896–E4905. 10.1073/pnas.1411117111 25355904 PMC4234577

[bib45] Nicot, A.S., H. Fares, B. Payrastre, A.D. Chisholm, M. Labouesse, and J. Laporte. 2006. The phosphoinositide kinase PIKfyve/Fab1p regulates terminal lysosome maturation in Caenorhabditis elegans. Mol. Biol. Cell. 17:3062–3074. 10.1091/mbc.e05-12-1120 16801682 PMC1483040

[bib46] Paschke, P., D.A. Knecht, A. Silale, D. Traynor, T.D. Williams, P.A. Thomason, R.H. Insall, J.R. Chubb, R.R. Kay, and D.M. Veltman. 2018. Rapid and efficient genetic engineering of both wild type and axenic strains of Dictyostelium discoideum. PLoS One. 13:e0196809. 10.1371/journal.pone.0196809 29847546 PMC5976153

[bib47] Qiao, Y., J.E. Choi, J.C. Tien, S.A. Simko, T. Rajendiran, J.N. Vo, A.D. Delekta, L. Wang, L. Xiao, N.B. Hodge, . 2021. Autophagy inhibition by targeting PIKfyve potentiates response to immune checkpoint blockade in prostate cancer. Nat. Cancer. 2:978–993. 10.1038/s43018-021-00237-1 34738088 PMC8562569

[bib48] Ratner, D.I., and P.C. Newell. 1978. Linkage analysis in Dictyostelium discoideum using multiply marked tester strains: Establishment of linkage group VII and the reassessment of earlier linkage data. J. Gen. Microbiol. 109:225–236. 10.1099/00221287-109-2-225 745003

[bib49] Rudge, S.A., D.M. Anderson, and S.D. Emr. 2004. Vacuole size control: Regulation of PtdIns(3,5)P2 levels by the vacuole-associated vac14-Fig4 complex, a PtdIns(3,5)P2-specific phosphatase. Mol. Biol. Cell. 15:24–36. 10.1091/mbc.e03-05-0297 14528018 PMC307524

[bib50] Rusten, T.E., L.M. Rodahl, K. Pattni, C. Englund, C. Samakovlis, S. Dove, A. Brech, and H. Stenmark. 2006. Fab1 phosphatidylinositol 3-phosphate 5-kinase controls trafficking but not silencing of endocytosed receptors. Mol. Biol. Cell. 17:3989–4001. 10.1091/mbc.e06-03-0239 16837550 PMC1556381

[bib51] Samie, M., X. Wang, X. Zhang, A. Goschka, X. Li, X. Cheng, E. Gregg, M. Azar, Y. Zhuo, A.G. Garrity, . 2013. A TRP channel in the lysosome regulates large particle phagocytosis via focal exocytosis. Dev. Cell. 26:511–524. 10.1016/j.devcel.2013.08.003 23993788 PMC3794471

[bib52] Sattler, N., R. Monroy, and T. Soldati. 2013. Quantitative analysis of phagocytosis and phagosome maturation. Methods Mol. Biol. 983:383–402. 10.1007/978-1-62703-302-2_21 23494319

[bib53] Sbrissa, D., O.C. Ikonomov, Z. Fu, T. Ijuin, J. Gruenberg, T. Takenawa, and A. Shisheva. 2007. Core protein machinery for mammalian phosphatidylinositol 3,5-bisphosphate synthesis and turnover that regulates the progression of endosomal transport. Novel Sac phosphatase joins the ArPIKfyve-PIKfyve complex. J. Biol. Chem. 282:23878–23891. 10.1074/jbc.M611678200 17556371

[bib54] Sbrissa, D., O.C. Ikonomov, and A. Shisheva. 1999. PIKfyve, a mammalian ortholog of yeast Fab1p lipid kinase, synthesizes 5-phosphoinositides. Effect of insulin. J. Biol. Chem. 274:21589–21597. 10.1074/jbc.274.31.21589 10419465

[bib55] Sbrissa, D., O.C. Ikonomov, and A. Shisheva. 2000. PIKfyve lipid kinase is a protein kinase: Downregulation of 5′-phosphoinositide product formation by autophosphorylation. Biochemistry. 39:15980–15989. 10.1021/bi001897f 11123925

[bib56] Shisheva, A. 2012. PIKfyve and its Lipid products in health and in sickness. Curr. Top. Microbiol. Immunol. 362:127–162. 10.1007/978-94-007-5025-8_7 23086417

[bib57] Sonn-Segev, A., K. Belacic, T. Bodrug, G. Young, R.T. VanderLinden, B.A. Schulman, J. Schimpf, T. Friedrich, P.V. Dip, T.U. Schwartz, . 2020. Quantifying the heterogeneity of macromolecular machines by mass photometry. Nat. Commun. 11:1772. 10.1038/s41467-020-15642-w 32286308 PMC7156492

[bib58] Veltman, D.M., G. Akar, L. Bosgraaf, and P.J. Van Haastert. 2009. A new set of small, extrachromosomal expression vectors for Dictyostelium discoideum. Plasmid. 61:110–118. 10.1016/j.plasmid.2008.11.003 19063918

[bib59] Wang, X., X. Zhang, X.P. Dong, M. Samie, X. Li, X. Cheng, A. Goschka, D. Shen, Y. Zhou, J. Harlow, . 2012. TPC proteins are phosphoinositide- activated sodium-selective ion channels in endosomes and lysosomes. Cell. 151:372–383. 10.1016/j.cell.2012.08.036 23063126 PMC3475186

[bib60] Wiegand, S., J. Kruse, S. Gronemann, and C. Hammann. 2011. Efficient generation of gene knockout plasmids for Dictyostelium discoideum using one-step cloning. Genomics. 97:321–325. 10.1016/j.ygeno.2011.02.001 21316445

[bib61] Wilczynska, Z., and P.R. Fisher. 1994. Analysis of a complex plasmid insertion in a phototaxis-deficient transformant of Dictyostelium discoideum selected on a Micrococcus luteus lawn. Plasmid. 32:182–194. 10.1006/plas.1994.1054 7846143

[bib62] Yamamoto, A., D.B. DeWald, I.V. Boronenkov, R.A. Anderson, S.D. Emr, and D. Koshland. 1995. Novel PI(4)P 5-kinase homologue, Fab1p, essential for normal vacuole function and morphology in yeast. Mol. Biol. Cell. 6:525–539. 10.1091/mbc.6.5.525 7663021 PMC301213

