# Supplementary figures and images for: A PI(3,5)P2 reporter reveals PIKfyve activity and dynamics on macropinosomes and phagosomes
Source: J Cell Biol. 2023 Jun 29;222(9):e202209077. doi: 10.1083/jcb.202209077 (PMC10309190; doi:10.1083/jcb.202209077)

**E**

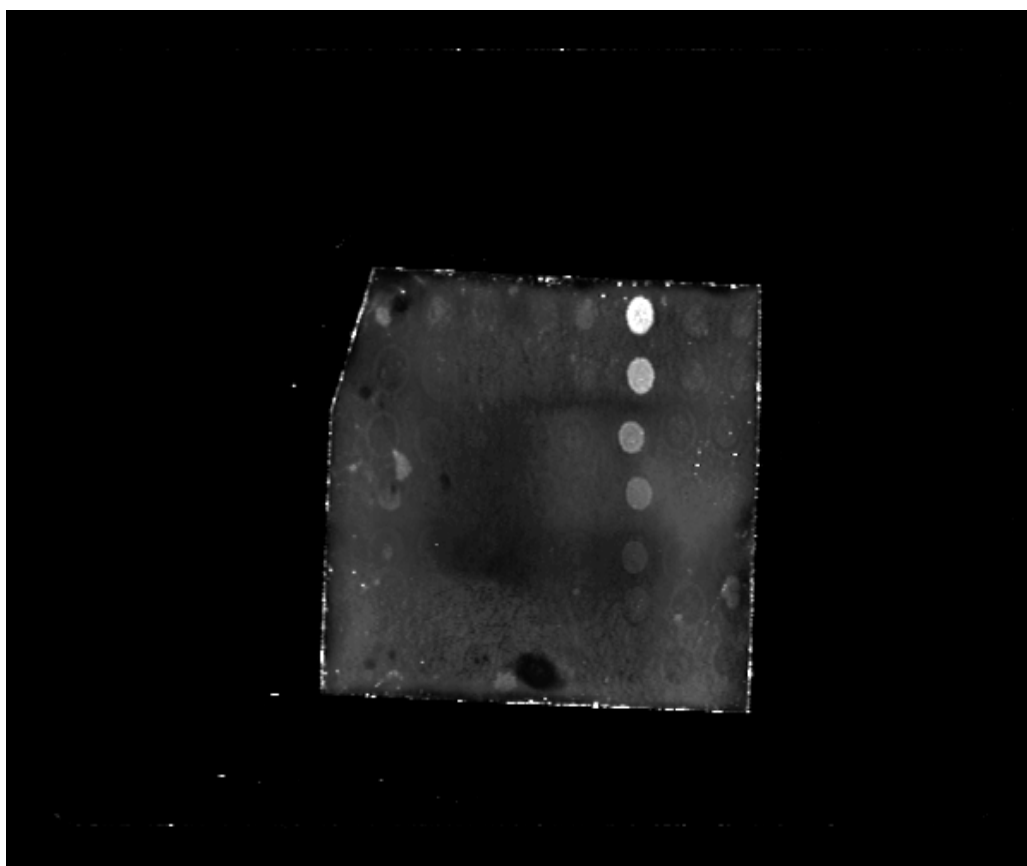

**Source data for Figure S1**

Supplement: SourceData F1 — is the source file for Fig. 1. [file JCB_202209077_SourceDataF1.pdf]

**A**

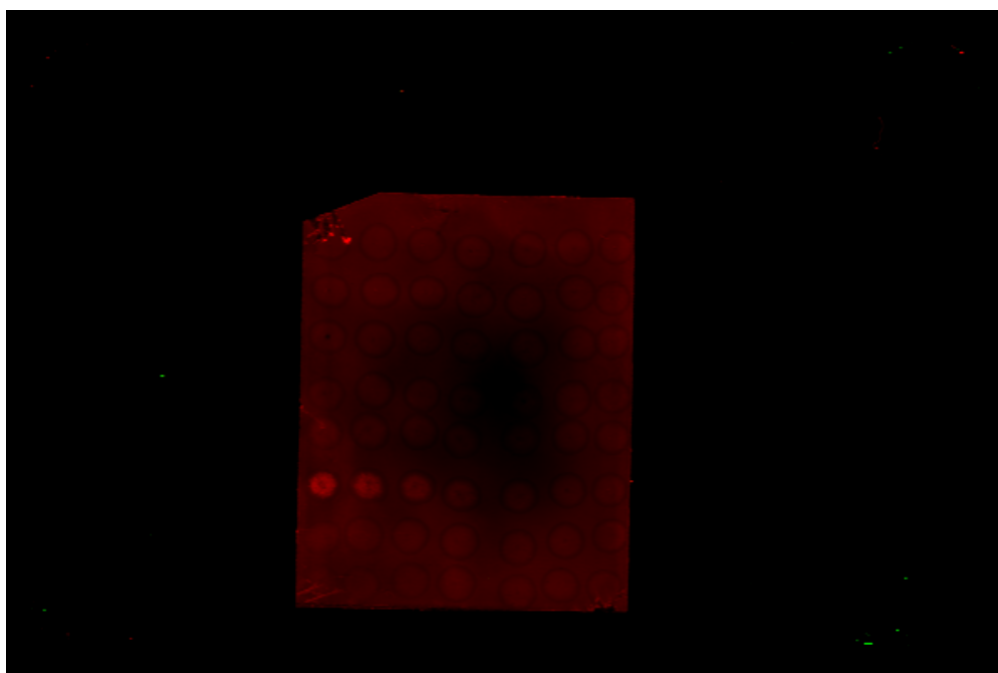

**B**

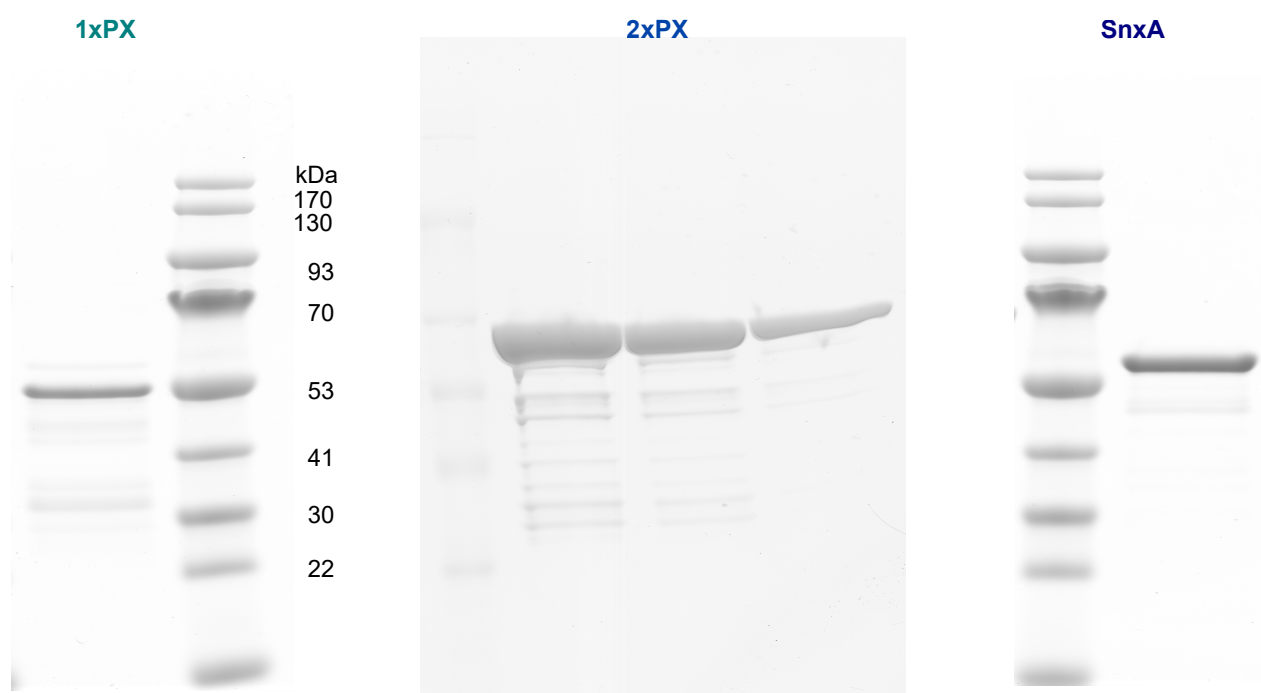

**Source data for Figure S1**

Supplement: SourceData FS1 — is the source file for Fig. S1. [file JCB_202209077_SourceDataFS1.pdf]
